# Differential genomics and transcriptomics between tyrosine kinase inhibitor-sensitive and -resistant BCR-ABL-dependent chronic myeloid leukemia

**DOI:** 10.18632/oncotarget.25752

**Published:** 2018-07-13

**Authors:** Neetu Singh, Anil Kumar Tripathi, Dinesh Kumar Sahu, Archana Mishra, Margaret Linan, Bianca Argente, Julia Varkey, Niranjan Parida, Rebecca Chowdhry, Hari Shyam, Nawazish Alam, Shivani Dixit, Pratap Shankar, Abhishek Mishra, Avinash Agarwal, Chris Yoo, Madan Lal Brahma Bhatt, Ravi Kant

**Affiliations:** ^1^ Molecular Biology Unit, Center for Advance Research, King George's Medical University, Lucknow, India; ^2^ Department of Clinical Hematology, King George's Medical University, Lucknow, India; ^3^ Department of Cardio Thoracic and Vascular Surgery, King George's Medical University, Lucknow, India; ^4^ Systems Imagination, Scottsdale, Arizona, USA; ^5^ Department of Periodontics, King George's Medical University, Lucknow, India; ^6^ Department of Medicine, King George's Medical University, Lucknow, India; ^7^ Department of Radiotherapy, King George's Medical University, Lucknow, India; ^8^ All India Institute of Medical Sciences, Rishikesh, India

**Keywords:** tyrosine kinase inhibitors, chronic myeloid leukemia, molecular-inversion-probe based array, human-transcriptome array 2.0, axiom biobank array

## Abstract

Previously, it has been stated that the BCR-ABL fusion-protein is sufficient to induce Chronic Myeloid Leukemia (CML), but additional genomic-changes are required for disease progression. Hence, we profiled control and tyrosine kinase inhibitors (TKI) alone or in combination with other drug-treated CML-samples in different phases, categorized as drug-sensitive and drug-resistant on the basis of BCR-ABL transcripts, the marker of major molecular-response. Molecular-profiling was done using the molecular-inversion probe-based-array, Human Transcriptomics-Array2.0, and Axiom-Biobank genotyping-arrays. At the transcript-level, clusters of control, TKI-resistant and TKI-sensitive cases were correlated with BCR-ABL transcript-levels. Both at the gene- and exon-levels, up-regulation of MPO, TPX2, and TYMS and down-regulation of STAT6, FOS, TGFBR2, and ITK lead up-regulation of the cell-cycle, DNA-replication, DNA-repair pathways and down-regulation of the immune-system, chemokine- and interleukin-signaling, TCR, TGF beta and MAPK signaling pathways. A comparison between TKI-sensitive and TKI-resistant cases revealed up-regulation of LAPTM4B, HLTF, PIEZO2, CFH, CD109, ANGPT1 in CML-resistant cases, leading to up-regulation of autophagy-, protein-ubiquitination-, stem-cell-, complement-, TGFβ- and homeostasis-pathways with specific involvement of the Tie2 and Basigin signaling-pathway. Dysregulated pathways were accompanied with low CNVs in CP-new and CP-UT-TKI-sensitive-cases with undetectable BCR-ABL-copies. High CNVs (previously reported gain of 9q34) were observed in BCR-ABL-independent and -dependent TKI, non-sensitive-CP-UT/AP-UT/B-UT and B-new samples. Further, genotyping CML-CP-UT cases with BCR-ABL 0-to-77.02%-copies, the identified, rsID239798 and rsID9475077, were associated with FAM83B, a candidate for therapeutic resistance. The presence of BCR-ABL, additional genetic-events, dysregulated-signaling-pathways and rsIDs associated with FAM83B in TKI-resistant-cases can be used to develop a signature-profile that may help in monitoring therapy.

## INTRODUCTION

Response to tyrosine kinase inhibitors (TKI) is usually monitored by measurement of hematologic, cytogenetic, and molecular responses [1, 2]. BCR-ABL mRNA transcripts are the major parameter used to assess the TKI molecular response and are usually measured in the peripheral blood at diagnosis, every 3 months until BCR-ABL transcripts are <0.1%, and then every 3-6 months thereafter [3]. According to the National Comprehensive Cancer Network (NCCN), Imatinib therapy is working if BCR-ABL transcripts are ≤10% after 3 months, <1% after 6 months, or undetectable after 18 months of therapy.

However, TKI-treated patients in which the BCR-ABL gene is no longer found or is un-detacted copies do not seem to be cured. In most of the cases, either the CML moves towards to the advanced phase, or BCR-ABL and CML cells show remission in more than half of the people who cease TKI treatment [4, 5].

Hence, genome-wide profiling of different phases of Imatinib-treated CML is expected to uncover signaling pathways and molecular mechanisms involved in Imatinib treatment at different phases of CML. Recent studies have suggested that clonal Copy Number Aberrations (CNAs) are rare or even absent in pediatric/adult-CML-chronic phase (CML-CP) and are relatively common at progressed stages [7, 12–14]. At the transcript level, signature genes identified in whole blood and leukemic stem cells have been shown to distinguish chronic phase (CP) from blast crisis (BC) [6] and to predict major cytogenetic response and non-response in chronic-phase CML patients treated with Imatinib [7]. However, an *in vitro* study and found no alteration in genomic changes of bone marrow-derived HSCs and HPCs from CML patients on Imatinib treatment [8].

Activation of ERK/MAPK, JAK-STAT, ErbB, cell surface genes, genes of oxidative metabolism and DNA repair pathways, activation of inflammatory cytokines and dysregulation of key cancer signaling pathways, as well as down-regulation of pro-differentiation and TGF-β/BMP signaling pathways have also been responsible for proliferation in CML [8–10].

In addition to copy number variations (CNVs) and expression profiling, genome-wide scoring of SNPs in different phases of Imatinib-treated CML will further help us to understand the resistance mechanism to TKIs.

At the transcript level, we were able to cluster TKI-sensitive and TKI-resistant cases and, after comparing, we identified the up-regulation of autophagy, complement, Tie-2 and Basigin signaling mediated homeostasis, protein ubiquitination, stem cell and down-regulation of immune system and TGF-beta pathways. Deregulation of these pathways was accompanied by low CNVs in CP-new and CP-UT-TKI-sensitive cases with undetectable BCR-ABL copies. High CNVs (previously reported gain of 9q34) were observed in BCR-ABL-independent and -dependent TKI, non-sensitive-CP-UT/AP-UT/B-UT and B-new samples. Further, using genotyping arrays, we assessed associations between individual SNPs and CML-resistance risk using odds ratios (ORs) and 95% confidence intervals (CIs) derived from logistic regression models. We identified that rsID239798 and 9475077 associated with the FAM83B gene, which may be directly related to treatment resistance in Imatinib-treated unrelated CML cases versus controls.

This analysis will be useful for a large segment of the medical research community for clinical screening of TKI-resistant and TKI-sensitive CML cases and develop a signature profile, which may help in monitoring therapy.

## RESULTS

### Differential gene expression levels among 35 CML-samples

**Figure 1:**
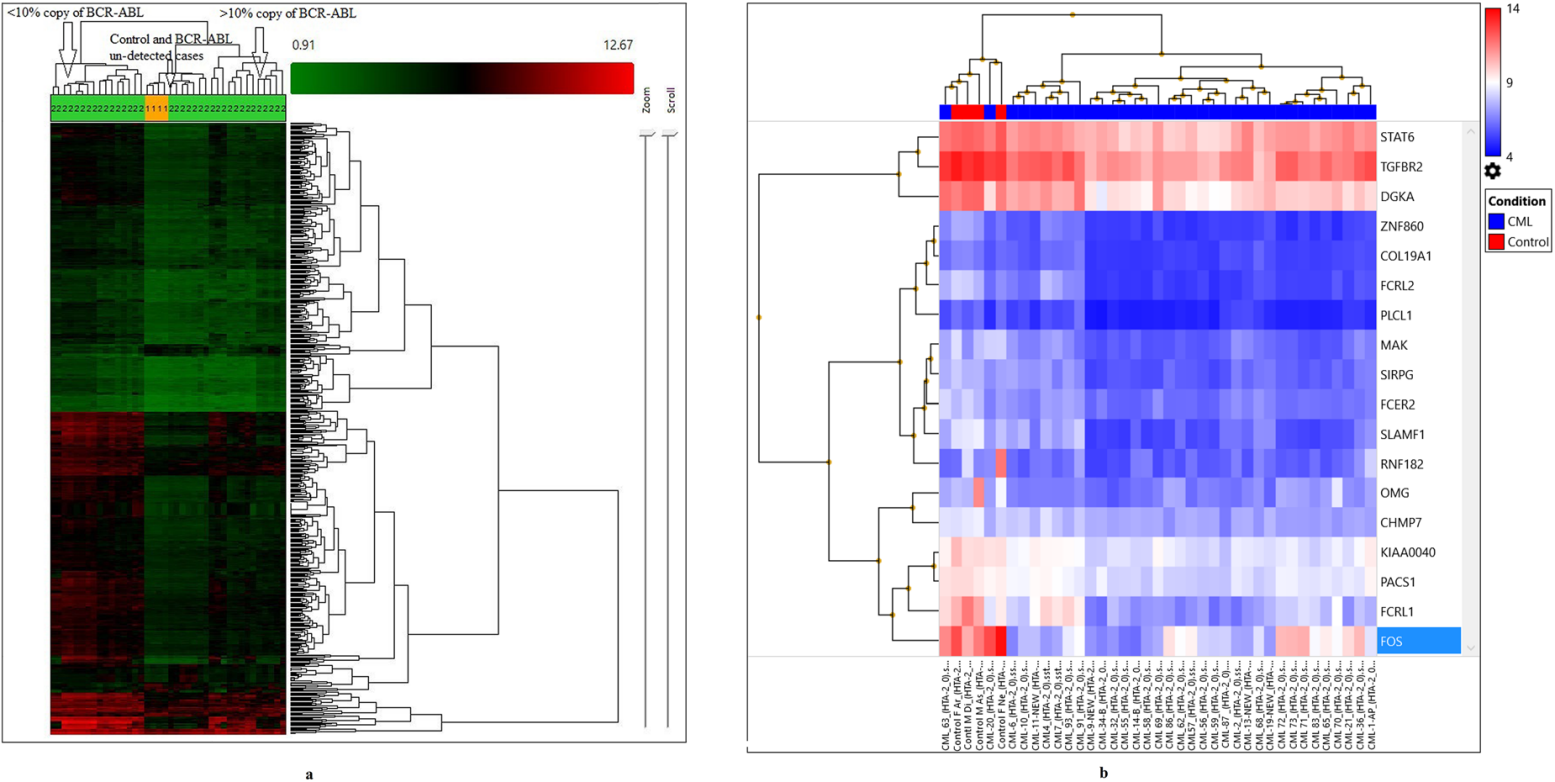
**(a)** Hierarchical clustering of the gene-level data revealed a distinct clustering of subgroups of all CML cases (35) and control (4) using default filtering criteria (fold-change (linear) < -2 or fold-change (linear) > 2 and ANOVA p value ≤0.05). **(b)** Gene level differential expression between 4 control and 35 CML samples using strict criteria (fold-change=2, p=0.001, and FDR p value=0.05) and considering only NM IDs, a highly significant down-regulation of 18 genes based upon which three clusters were formed.

To identify significant differential gene expression levels between 4 control and 35 CML samples (including both TKI-treated and newly diagnosed cases), a one-way between-subjects ANOVA algorithm was used. Differentially expressed coding and non-coding transcript clusters were identified using default filtering criteria (fold-change (linear) < -2 or fold-change (linear) > 2 and ANOVA p value ≤0.05). The array that was used measures 67,528 genes, including both coding (44,699) and non-coding (22,829) genes. Out of the total number of genes, only 2,073 genes were differentially expressed (1,425 coding and 648 non-coding). Compared to control among all CML samples, 69 genes were up-regulated (49 coding and 20 non-coding), and 2,004 genes were down-regulated (1,376 coding and 628 non-coding). Hierarchical clustering of the gene-level data revealed distinct clustering of 35 CML samples, including tri-phasic-TKI-treated, new cases and four normal controls (p=0.01, Figure 1a, Table 1 ). When comparing clusters with copies of BCR-ABL, samples with un-detected copies of BCR-ABL (CP-UT, AP-UT and some of CP-new cases) were classified under the first cluster as non-sensitive cases (p=0.01). The second cluster-sub-cluster-I included all control samples, and the second cluster-sub-cluster-II included CP new cases and cases in which copies of BCR-ABL were undetectable. The third cluster included samples showing ≥1-10% copies of BCR-ABL (CP-UT, AP-UT and new blast cases) (Table 1).

**Table 1:** Hierarchical clustering of the gene-level data revealed distinct clustering of 35 samples of CML including all the three phases and 4 normal controls at p=0.01

Cluster-I-Sub-cluster-I								
Sample-ID	CML-Phases and treatment status	Age/gender	Sample collection date	Time of Assessment	Treatment	Date of BCR-ABL assessment	BCR-ABL percentage	Comments based on BCR-ABL and treatment
CML-69	AP-UT-69	40/F	16-04-2015	3 months	Imatinib	04-11-2014	14.08	Imatinib non-sensitive
						29-04-2015	9.75	
CML-83	CP-New-83	70/M	27-04-2015	New	Imatinib	18-04-2015	77.02	Not known
CML-86	AP-UT-86	49/M	27-04-2015	5 months	Imatinib and march 2015 Nilotinib	25-11-2014	18.04	Imatinib non-sensitive
						01-05-2015	2.45	
CML-72	AP-UT-72	45/M	16-04-2015	6 months	Imatinib and june 2015 Nilotinib	16-10-2014	25.19	Imatinib non-sensitive and Nilotinib sensitive (TKI-sensitive)
						01-05-2015	5.45	
CML-73	CP-New-73	50/M	22-04-2015	New	Imatinib	25-04-2015	73.8	Not known
CML-70	AP-UT-70	30/M	16-04-2015	1.0 year	Imatinib and december 2014 nilotinib	10-01-2015	20	Imatinib and Nilotinib non-sensitive(TKI-non sensitive)
						22-04-2015	1.63	
CML-71	CP-UT-71	52/M	16-04-2015	8.2 yrs	Initially Hydab, Unidrea and since october 2012 Imatinib and switched to nilotinib December 2014	15-04-2014	20	Initially Hydab, Unidrea and since october 2012 Imatinib and switched to nilotinib December 2014, TKI-non-sensitive
						22-06-2015	8.63	
**Cluster-I-Sub-cluster-II**								
CML-55	CP-UT-55	60/M	06-02-2015	3 months	Hydroxyurea, Zyloric and Imatinib	02-12-2014	98.47	Hydroxyurea, Zyloric and Imatinib non- sensitive
						01-05-2015	9.75	
CML-56	CP-UT-56	43/F	09-02-2015	1.0 yr	Hydroxyurea, Zyloric and Imatinib	29-02-2014	89.5	Hydroxyurea, Zyloric and Imatinib non-sensitive
						24-03-2015	1.2	
CML-58	CP-UT-58	32/F	09-02-2015	9 months	Hydroxyurea, Zyloric and Imatinib	12-04-2015	58.75	Hydroxyurea, Zyloric and Imatinib non-sensitive
						12-04-2015	9.8	
CML-62	AP-UT-62	42/M	09-04-2015	4.4 yrs	Unidrea, Imatinib and since February 2014 Nilotinib	06-06-2014	14.96	Uridrea, Imatinib and Nilotinib non-sensitive (TKI-non-sensitive)
						22-06-2015	4.03	
CML-65	CP-UT-65	43/M	20-04-2015	7.9 yrs	Hydroxyurea, Zyloric and Imatinib later Nilotinib	24-06-2014	18.95	Hydroxyurea, Zyloric Imatinib and Nilotinib was sensitive (TKI-non-sensitive)
						22-04-2015	4.63	
CML-57	CP-UT-57	42/F	09-02-2015	7 months	Hydroxyurea, Zyloric and Imatinib	22-07-2014	67.45	Hydroxyurea, Zyloric and Imatinib non-sensitive
						22-04-2015	8	
CML-59	CP-UT-59	15/M	01-04-2015	3 months	Hydroxyurea, Zyloric and Imatinib	11-12-2014	15.75	Hydroxyurea, Zyloric and Imatinib non-sensitive
						29-04-2015	9.75	
CML-14	B-New-14	35/F	29-05-2014	New	Hydroxyurea, Zyloric and Imatinib	09-06-2014	85.56	Not known
All controls								
**Cluster-2-sub cluster-II**								
CML-10	CP-New-10	20/M	28-05-2014	New	Imatinib	28-05-2014	120	Not known
						06-02-2015	0.95	
CML-07	CP-UT-07	41/M	22-05-2014	7.2 yrs	Imatinib	28-02-2007	100%	Imatinib sensitive
						22-07-2014	not detected	
CML-6	CP-UT-6	24/M	22-05-2014	7 months	Imatinib	21-11-2013	75	Imatinib sensitive
						17-06-2014	not detected	
CML-04	CP-UT-4	14/M	22-05-2014	1.2 year	Imatinib	15-03-2013	100	Imatinib sensitive
						06-09-2014	not detected	
CML-02	CP-UT-2	50/M	22-05-2014	4 yrs	Initially with Droxygel (Antacid), Unidrea and later with Imatinib	14-02-2013	100%	Initially with Droxygel (Antacid), Unidrea and later with Imatinib-sensitive
						20-03-2014	not detected	
CML-1	AP-UT-1	35/M	22-05-2014	1.8 yrs	Imatinib	30-08-2012	26	Imatinib sensitive
CML-20	CP-UT-20	33/F	05-06-2014	1 month	Imatinib	21-05-2014	97.54	NA
**Cluster-3-sub cluster-I**								
CML-34	B-New-34	24/M	12-06-2014	New	Imatinib and since February 2015 Nilotinib	20-06-2014	35.63	Imatinib non-sensitive
						27-11-2014	95.37	
CML-09	CP-New-9	46/M	28-05-2014	New	Imatinib	28-05-2014	100	Not known
CML-32	AP-UT-32	27/F	12-06-2014	15 yrs	Initially treated with Myeleron, Hydab, Zyloric since may 2005 on Imatinib	13-07-2014	55.63	Initially treated with Myeleron, Hydab, Zyloric since may 2005 on Imatinib-non-sensitive
						17-11-2014	35.37	
**Cluster-3-sub cluster-II**								
CML-13	CP-UT-13	50/M	29-05-2014	7.1 yrs	Droxygel (Antacid), Unidrea and Imatinib	20-02-2014	30	Droxygel (Antacid), Unidrea and Imatinib sensitive
						12-03-2015	0.1	
CML-21	CP-UT-21	60/M	05-06-2014	3.8 yrs	Droxygel (Antacid), Unidrea and Imatinib	14-11-2013	75	Droxygel (Antacid), Unidrea and Imatinib sensitive
						31-07-2014	0.11	
CML-19	B-New-19	28/M	29-05-2014	New	Imatinib	15-05-2014	100	Not known
CML-11	CP-UT-11	33/F	28-05-2014	4.2 yrs	Hydab and Imatinib	21-04-2010	97.54	Hydab and Imatinib sensitive
						21-05-2014		Not detectable
CML-36	CP-UT-36	23/M	03-07-2014	6.10 yrs	Initially Hydab and presently on Imatinib	20-02-2010	30	Initially Hydab and Imatinib sensitive
						30-10-2014	0.11	
**Cluster-3-sub cluster-III**								
CML-93	CP-UT-93	60/M	08-06-2015	11 months	Imatinib	14-05-2014	100	Imatinib non-sensitive
						11-03-2015	70.13	
CML-68	CP-UT-68	28/M	16-04-2015	4 months	Imatinib	22-09-2014	45.2	Imatinib non-sensitive
						22-04-2015	16.1	
CML-91	CP-UT-91	66/F	08-06-2015	6 months	Imatinib	10-09-2014	38.52	Imatinib non-sensitive
						22-04-2015	26.1	
CML-63	CP-UT-63	37/M	09-04-2015	7 months	Imatinib	21-08-2014	100	Imatinib non-sensitive
						09-04-2015	80	
CML-87	CP-UT-87	45/F	08-06-2015	2.9 yrs	Imatinib	01-07-2015	86	Imatinib non-sensitive

Both sub-cluster-I and II of I-cluster included non-sensitive cases with un-detected copies of BCR-ABL samples (Chronic Phase under treatment; CP-UT, accelerated phase under treatment; AP-UT and some of Chronic Phase-new; CP-new cases) In cluster-I sub-cluster-II all were treated with Hydroxyurea, Zyloric and Imatinib and were non-sensitive to the treatment. The second cluster-sub-cluster-I included all control samples and second cluster-sub-cluster-II included CP-new cases and cases where copies of BCR-ABL were undetectable. Third cluster included samples showing > or = 1 to 10% copies of BCR-ABL samples (CP-UT, AP-UT and new blast cases). In cluster-3 sub-cluster-I one patient was initially treated with Myeleron (Busulfan-15 years back), Hydab, Zyloric and later with Imatinib and in cluster-3 sub-cluster-II the samples except CML-19 all other were treated with Droxygel (Antacid), Unidrea/Hydab and Imatinib.

In the first cluster-sub-cluster-II, the CML-55, 56, 57, 58, 59 samples were all from patients treated with Hydroxyurea, Zyloric and Imatinib and were non-sensitive to treatment but for shorter time period (all within one year). In the third cluster sub-cluster-I, one patient was initially treated with Myeleron (Busulfan 15 years earlier), Hydab, and Zyloric and then later with Imatinib and then developed resistance. The third cluster sub-cluster-II contained samples treated with Droxygel (Antacid), Unidrea/Hydab and Imatinib for a longer time period (3.8 years-7.1 years).

Supplementary Table 1 shows the differential expression of genes between 4 control and 35 CML samples, including both TKI-treated and fresh diagnosed cases (p=0.01, fold-change=2, default FDR p value). These differentially expressed genes showed highly significant involvement with the Retinoblastoma (RB) (24 up-regulated and 1 down-regulated), cell cycle (14 up-regulated and 2 down-regulated), DNA replication (8 up-regulated), DNA IR-damage and cellular response via ATR (9 up-regulated and 1 down-regulated), allograft rejection (10 down-regulated), T cell antigen receptor (TCR) signaling pathway (9 down-regulated), Vitamin D receptor pathway (12 down-regulated), histone modifications (7 up-regulated), gastric cancer networks 1 and 2 (5 and 6 genes up-regulated, respectively), G1 to S cell cycle control (6 up-regulated and 1 down-regulated), mitotic G1-G1/S phases (12 up-regulated), spinal cord injury (2 up-regulated and 6 down-regulated), hair follicle development: cyto-differentiation (1 up-regulated and 6 down-regulated) and TGF-beta signaling pathways (2 up-regulated and 6 down-regulated, Supplementary Table 2a).

With strict criteria (fold-change=2, p=0.001, and FDR p value=0.05) and considering only NM IDs on gene level differential expression between 4 control and 35 CML samples, a highly significant down-regulation of 18 genes was identified upon which the following three clusters were formed: the first cluster included control samples CML-20 and CML-63; the second cluster included CML-4, 7, 91, 93, 1, 11, 19, 6 and 10; and the third cluster included three sub-clusters-I (21, 36, 72, 73, 71, 83, 65, and 70), sub-cluster-II (32, 55, 9, 58, 14, 34, and 69) and sub-cluster-III (56, 59, 86, 62, 57, 2, 13, 68, and 87, Figure 1b).

At a significance level of p=0.001 and an FDR p value=0.05, 18 genes showed significant down-regulation (p=0.05) among 36 pathways in CML, including the highly significant PDGFR-beta pathway (significance of 3.77 at p=0.000171), the TGF-beta receptor signaling pathway (significance of 3.21 at p=0.000618), the spinal cord injury pathway (significance of 2.56 at p=0.002758), the TGF-beta signaling pathway (significance of 2.44 at p=0.003649), and the MAPK signaling pathway (significance of 1.94 at p=0.011546, Supplementary Table 2b).

### Exon-specific expression among control and 35-CML samples

Relative exon-specific expression was measured between two conditions (control and all 35-CML samples) after excluding gene level data and passing through default filtering criteria (Splicing Index (linear) < -2 or Splicing Index (linear) > 2, ANOVA p value < 0.05, a gene must be expressed in both conditions, a PSR/Junction must be expressed in at least one condition, and a gene must contain at least one PSR) using the following algorithms: 1.) Splicing Index; 2.) one-way between-subjects ANOVA (unpaired); 3.) false discovery rate < 0.05; 4.) use an eligible PSR to determine gene expression if it presents in >=50% of all transcript isoforms; 5.) a gene is expressed in a sample if >=50% of its eligible PSRs have DABG p value < 0.05; 6.) a condition has this gene expressed if >=50% of its samples express this gene; and 7.) a PSR/Junction is expressed in a condition if >=50% of samples have DABG p value < 0.05 among the samples analyzed.

However, on applying strict criteria (Exon-Splicing Index (linear) < -10 or Splicing Index (linear) > 10, exon ANOVA p value < 0.001, exon FDR p value < 0.05, fold-change < -10 or > 10 for genes expressed in both conditions), we observed 7.59-, 15.22- and 5.09-fold down-regulation of IL-2-inducible T cell kinase (ITK-exon SI -10.28, exon p value=0.0000123, exon FDR p value=0.0201), FBJ murine osteosarcoma viral oncogene homolog (FOS-exon SI -12.57, exon p value=0.000000587, exon FDR p value=0.006), and src kinase associated phosphoprotein 1 (SKAP1-exon SI -10.37, exon p value= 0.0000354, exon FDR p value=0.031), respectively, among CML samples. However, Myeloperoxidase (MPO-exon SI -30.08, exon p value=0.0001, exon FDR p value=0.044), Thymidylate synthetase (TYMS-exon SI= -15.08, exon p value=0.0000896, exon FDR p value=0.044) and TPX2, microtubule-associated (TPX2-exon SI=13.79, exon p value= 0.0001, exon FDR p value=0.046) showed up-regulation of 121.81-, 10.12- and 7.56-fold, respectively, among CML cases. After submitting these genes to the Reactome Pathway database, we identified ITK, FOS and SKAP1 mediated down-regulation of the immune system (Supplementary Table 3). MPO, TPX2, TYMS specifically up-regulated cell cycle pathways and individually, TPX2 mediated the up-regulation of phosphorylation altering the transcriptional regulation of TP53 activity and TYMS-related G1/S-Specific transcription through interconversion of nucleotide di- and triphosphates. Further, MPO enhanced neutrophil degranulation, thereby affecting the innate immune system (Supplementary Table 3).

### Copy number variation profiling of 34 CML-samples

CNV profiling was completed for 39 CML-samples (34 CML and five control samples), and the results were analyzed using Nexus version 7.5 (Biodiscovery, Inc. CA USA). Samples were further categorized on the basis of CNVs, percent loss of heterozygosity (LOH) and percent genome change (Table 2a). In group I, CP-new and B-New cases (CML9, 10, 14) and CP-UT cases with un-detected BCR-ABL levels (CML56, 57, 58, CML55, 59) showed low copy number variation, i.e., there were no significant gains, but there were losses of HOXA9, HOXA11, HOXA13 (7p15.2) and CDK4 (12q14.1, Table 2b and 2c). In group II, high CNVs were observed in both samples with undetectable and <20% BCR-ABL copies, including both sensitive and non-sensitive cases and >20% BCR-ABL copies (AP-UT-22, 37, 38 and B-New-34, B-UT-47, 48)] (Table 2b). On aggregate analysis, the high CNVs-TKIs non-sensitive group showed significant gains of SDHB (1p36.13), FGFR3, WHSC1 (4p16.3), FNBP1, ABL1, NUP214, TSC1, RALGDS (9q34.11 - q34.2), YWHAE (17p13.3), CDK12 (17q12) and U2AF1 (21q22.3), with some important losses as listed in Table 2c.

**Table 2a:** Percent genome change identified in chronic myeloid leukemia samples of different phases undergoing treatment (Chronic Phase under treatment; CP-UT, accelerated phase under treatment; AP-UT and Blast Phase under treatment; B-UT ) and new cases (Chronic Phase-new; CP-new and Blast new; B-new) cases through copy number and somatic mutation related molecular inversion probe based array

	Sample	Quality	Total CN aberrations	% LOH	% Genome Changed	OS-MAPD	OS-ndSNPQC	OS-CelPair Check Status	OS-nd WavinessSd	OS-% Aberr. Cells	OS-Ploidy	OS-Low Diploid Flag
1	CML-09	1.57E-01	62	7.55E-01	4.23E-01	2.69E-01	3.30E+01	Pass	1.90E-01	homogeneous	2.00E+00	No
2	CML-10	4.92E-01	60	3.42E+01	3.53E+00	4.68E-01	1.01E+01	Pass	2.16E-01	NA	NaN	Yes
3	CML-14	1.60E-01	34	2.61E+00	2.88E-01	2.69E-01	3.17E+01	Pass	1.38E-01	homogeneous	2.00E+00	No
4	CML-55	3.64E-01	79	8.32E+00	1.20E+01	3.91E-01	1.31E+01	Pass	8.74E-02	NA	NaN	No
5	CML-56	1.97E-01	91	3.30E+00	2.66E+00	3.03E-01	1.23E+01	Pass	1.59E-01	NA	NaN	No
6	CML-57	1.51E-01	80	2.73E+00	1.41E+00	2.68E-01	1.12E+01	Pass	1.36E-01	NA	NaN	No
7	CML-58	1.53E-01	118	5.50E+00	4.89E+00	2.69E-01	1.03E+01	Pass	1.37E-01	NA	NaN	No
8	CML-59	4.12E-01	97	1.13E+01	1.01E+01	4.35E-01	9.17E+00	Pass	1.87E-01	NA	NaN	No
9	CML-13	1.77E-01	99	2.01E+00	7.69E-01	2.84E-01	3.00E+01	Pass	2.34E-01	homogeneous	2.00E+00	No
10	CML-21	1.29E-01	81	7.30E-01	4.99E-01	2.42E-01	4.16E+01	Pass	1.53E-01	homogeneous	2.00E+00	No
11	CML-23	1.60E-01	191	3.56E+00	3.07E+00	2.58E-01	3.37E+01	Pass	2.47E-01	70	2.00E+00	No
12	CML-26	1.35E-01	52	1.34E+00	4.32E-01	2.50E-01	4.11E+01	Pass	1.40E-01	homogeneous	2.00E+00	No
13	CML-29	4.25E-01	63	1.29E+01	1.45E+01	4.44E-01	1.05E+01	Pass	1.09E-01	NA	NaN	No
14	CML-30	1.85E-01	226	2.77E+00	4.18E+00	2.78E-01	3.27E+01	Pass	2.68E-01	NA	NaN	No
15	CML-33	1.86E-01	172	5.18E+00	3.03E+00	2.83E-01	2.72E+01	Pass	2.18E-01	35	2.00E+00	No
16	CML-35	1.84E-01	106	4.45E+00	9.00E-01	2.80E-01	3.03E+01	Pass	2.54E-01	homogeneous	2.00E+00	No
17	CML-36	2.03E-01	255	7.45E+00	2.51E+00	2.93E-01	3.14E+01	Pass	3.14E-01	95	2.00E+00	No
18	CML-43	1.53E-01	106	1.56E+00	8.02E-01	2.61E-01	3.88E+01	Pass	1.83E-01	homogeneous	2.00E+00	No
19	CML-02	1.54E-01	84	1.86E+00	5.93E-01	2.61E-01	3.47E+01	Pass	2.23E-01	homogeneous	2.00E+00	No
20	CML-04	1.72E-01	183	3.43E+00	4.52E+00	2.74E-01	3.35E+01	Pass	2.47E-01	NA	NaN	No
21	CML-07	1.64E-01	82	1.98E+00	6.86E-01	2.77E-01	3.48E+01	Pass	2.06E-01	homogeneous	2.00E+00	No
22	CML-11	1.85E-01	158	5.40E+00	1.38E+00	2.83E-01	3.14E+01	Pass	2.92E-01	homogeneous	2.00E+00	No
23	CML-48	1.70E-01	186	3.22E+00	1.98E+00	2.71E-01	3.26E+01	Pass	2.36E-01	50	2.00E+00	No
24	CML-49	2.17E-01	205	6.64E+00	4.14E+00	3.12E-01	2.99E+01	Pass	2.89E-01	85	2.00E+00	No
25	CML-51	2.39E-01	166	4.62E+00	1.44E+00	3.29E-01	3.23E+01	Pass	2.80E-01	homogeneous	2.00E+00	No
26	CML-24	1.75E-01	295	4.62E+00	3.65E+00	2.69E-01	3.41E+01	Pass	2.84E-01	40	2.00E+00	No
27	CML-01	2.44E-01	197	9.40E+00	5.90E+00	3.18E-01	1.67E+01	Pass	2.74E-01	NA	NaN	No
28	CML-22	2.40E-01	86	3.80E+00	1.00E+01	3.31E-01	1.82E+01	Pass	1.49E-01	NA	NaN	No
29	CML-32	2.06E-01	241	7.83E+00	3.12E+00	2.89E-01	2.92E+01	Pass	2.98E-01	65	2.00E+00	No
30	CML-34	4.45E-01	231	1.09E+01	1.26E+01	4.60E-01	2.06E+01	Pass	2.83E-01	NA	NaN	No
31	CML-37	1.95E-01	135	6.61E+00	1.20E+00	2.83E-01	2.51E+01	Pass	2.73E-01	homogeneous	2.00E+00	No
32	CML-38	2.08E-01	353	5.44E+00	8.73E+00	3.01E-01	3.20E+01	Pass	3.11E-01	NA	NaN	No
33	CML-47	2.82E-01	153	3.53E+00	1.72E+00	3.56E-01	3.07E+01	Pass	2.86E-01	homogeneous	2.00E+00	No
34	CML- 8	1.94E-01	187	1.04E+01	5.75E+00	2.81E-01	3.04E+01	Pass	3.46E-01	85	2.00E+00	No

**Table 2b:** Nexus 7.5 analysed chronic myeloid leukemia samples of different undergoing treatment (Chronic Phase under treatment; CP-UT, accelerated phase under treatment; AP-UT and Blast Phase under treatment; B-UT ) and new cases (Chronic Phase-new; CP-new and Blast new; B-new) cases: clustered on the basis of copy number variations in relation to BCR-ABL transcript levels

Sample-ID	CML-Phases and treatment status	Age/Sex	Sample collection date	Time of Assessment	Treatment	Date	BCR-ABL%	Comments based on BCR-ABL
**Low CNVs group with undetectable BCR-ABL transcript levels, new cases or <10%**								
CML-09	CP-New-9	46/M	28-05-2014	New	Imatinib	28-05-2014	100	-
CML-14	B-New-14	35/F	29-05-2014	New	Hydroxyurea, Zyloric and Imatinib	09-06-2014	85.56	-
CML-10	CP-New-10	20/M	28-05-2014	New	Imatinib	28-05-2014	120	-
							0.95	
CML-55	CP-UT-55	60/M	06-02-2015	3 months	Hydroxyurea, Zyloric and Imatinib	02-12-2014	98.47	Hydroxyurea, Zyloric and Imatinib non-sensitive
						01-05-2015	9.75	
CML-56	CP-UT-56	43/F	09-02-2015	1.0 yr	Hydroxyurea, Zyloric and Imatinib	29-02-2014	89.5	Hydroxyurea, Zyloric and Imatinib non sensitive
						24-03-2015	1.2	
CML-57	CP-UT-57	42/F	09-02-2015	7 months	Hydroxyurea, Zyloric and Imatinib	22-07-2014	67.45	Hydroxyurea, Zyloric and Imatinib non sensitive
						22-04-2015	8	
CML-58	CP-UT-58	32/F	09-02-2015	9 months	Hydroxyurea, Zyloric and Imatinib	12-06-2014	58.75	Hydroxyurea, Zyloric and Imatinib sensitive
						12-04-2015	9.8	
CML-59	CP-UT-59	15/M	01-04-2015	3 months	Hydroxyurea, Zyloric and Imatinib	11-12-2014	15.75	Hydroxyurea, Zyloric and Imatinib sensitive
						29-04-2015	9.75	
**High CNVs in undetectable/<20% BCR-ABL transcript**								
CML-13	CP-UT-13	50/M	29-05-2014	7.1 yrs	Droxygel (Antacid), Unidrea and Imatinib	20-02-2014	30	Droxygel (Antacid), Unidrea and Imatinib sensitive
						12-03-2015	0.1	
CML-21	CP-UT-21	60/M	05-06-2014	3.8 yrs	Droxygel (Antacid), Unidrea and Imatinib	14-11-2013	75	Droxygel (Antacid), Unidrea and Imatinib sensitive
						31-07-2014	0.11	
CML-23	CP-UT-23	40/M	05-06-2014	2.4 yrs	Unidrea and Imatinib, Nilotinib	31-07-2013	28.35	Unidrea Imatinib and Nilotinib-non-sensitive
						13-05-2014	11.9	
CML-26	CP-UT-26	32/M	05-06-2014	2.1 yrs	Imatinib	26-03-2012	150	Imatinib non-sensitive
						10-05-2014	13.6	
CML-29	B-UT-29	35F	05-06-2014	1 month	Imatinib	04-05-2014	89.12	Imatinib non-sensitive
						01-08-2014	15.89	
CML-30	CP-New-30	22/M	12-06-2014	-	Imatinib	24-06-2014	11	Not known
						30-09-2014	12.65	
CML-33	AP-UT-33	20/M	28-06-2014	2 yrs	Imatinib	24-07-2012	100	Imatinib non-sensitive
						27-06-2014	12.18	
CML-35	AP-UT-35	28/F	03-07-2014	6.3 yrs	Imatinib	24-07-2010	100	Imatinib non-sensitive
						13-04-2013	0.02	
						03-07-2014	8.56	
CML-36	CP-UT-36	23/M	03-07-2014	6.10 yrs	Hydab and Imatinib	20-02-2010	30	Hydab and Imatinib sensitive
						30-10-2014	0.16	
CML-43	CP-UT-43	27/M	10-07-2014	7 yrs	Initially Hydroxyurea and Imatinib	23-02-2012	55.89	Hydroxyurea and Imatinib-sensitive
						17-06-2014	11.18	
						14-03-2015	0	
CML-24	CP-UT-24	26/F	05-06-2014	8.5 yrs	Initially treated with Myeleron, Hydab irocos, Zyloric since may 2005 on Imatinib	24-04-2014	30	Initially treated with Myeleron, Hydab irocos, Zyloric since may 2005 on Imatinib-non-sensitive
						21-05-2015	15.85	
CML-02	CP-UT-2	50/M	22-05-2014	4 yrs	Droxygel (Antacid), Unidrea and Imatinib	14-02-2013	100%	Droxygel (Antacid), Unidrea and Imatinib-sensitive
						20-03-2014	not detected	
CML-04	CP-UT-4	14/M	22-05-2014	1.2 yrs	Imatinib	15-07-2013	100	Imatinib sensitive
						06-09-2014	not detected	
CML-07	CP-UT-7	41/M	22-05-2014	7.2 yrs	Imatinib	28-02-2007	100%	Imatinib-sensitive
						22-07-2014	not detected	
CML-11	CP-UT-11	33/F	28-05-2014	4.2 yrs	Hydab and Imatinib	21-04-2010	97.54	Hydab and Imatinib sensitive
						21-05-2014	Not detectable	
CML-48	CP-UT48	33/M	31-07-2014	10.4 yrs	Initially Hydroxyurea and since December 2004 Imatinib	01-10-2004	100	Initially Hydroxyurea and since December 2004 Imatinib non-sensitive
						29-05-2014	9.94	
CML-49	Blast-UT-49	15/M	31-07-2014	1.4 yrs	Imatinib	06-03-2013	100	Imatinib non-sensitive
						30-06-2014	not detected	
CML-51	CP-UT-51	60/M	31-07-2014	25 yrs	Initially treated with Myeleron, Hydab irocos, Zyloric since may 2005 on Imatinib	12-05-2013	30	Initially treated with Myeleron, Hydab irocos, Zyloric since may 2005 on Imatinib non-sensitive
						11-06-2014	8.98	
CML-01	AP-UT-1	35/M	22-05-2014	1.8 yrs	Imatinib	30-08-2012	26	Imatinib sensitive
**High CNVs with BCR-ABL transcript levels >20%**								
CML-22	AP-UT-22	40/M	05-06-2014	5.2 yrs	Hydab and Imatinib	17-11-2013	39.07	Hydab and Imatinib non-sensitive
						05-08-2014	20.11	
CML-32	AP-UT-32	27/F	12-06-2014	15 yrs	Initially treated with Myeleron, Hydab irocos, Zyloric since may 2005 on Imatinib	13-07-2014	55.63	Initially treated with Myeleron, Hydab irocos, Zyloric since may 2005 on Imatinib-non-sensitive
						17-11-2014	35.37	
CML-34	B-New-34	24/M	12-06-2014	New	Imatinib and since February 2015 Nilotinib	20-06-2014	35.63	Imatinib and Nilotinib non-sensitive
						27-11-2014	95.37	
CML-37	AP-UT-37	27/F	03-07-2014	8 months	Imatinib	01-04-2013	100	Imatinib non-sensitive
						03-07-2014	50	
CML-38	AP-UT-38	60/M	03-07-2014	1.5 yrs	Imatinib	29-03-2013	79.01	Imatinib non-sensitive
						24-06-2014	35.12	
CML-47	B-UT-47	48/M	17-07-2014	3 months	Imatinib and 29-05-2014 Uridrea	27-07-2014	40.12	Unidrea and Imatinib non-sensitive
						29-10-2014	32.45	
CML-08	B-UT-8	24/M	22-05-2014	8 months	Imatinib	26-09-2013	100	Imatinib non-sensitive
						20-06-2014	26.89	

**Table 2C:** Aggregate analysis through Nexus 7.5 of low and high copy number variation group in relation to BCR-ABL levels

Low CNVs with undetectable BCR-ABL (TKIs-sensitive) group			
Cytoband Location	Event	P-Value	CancerGeneCensus-Sanger.txt
7p15.2	CN Loss	0.001	HOXA9, HOXA11, HOXA13
12q14.1	CN Loss	0.002	CDK4
**High CNVs (CN loss)-BCR-ABL dependent and independent (TKIs-non-sensitive) group**			
Cytoband Location	Event	P-Value	CancerGeneCensus-Sanger.txt
1p36.33 - p36.32	CN Loss	0.007	TNFRSF14
1p36.32	CN Loss	0.007	PRDM16
1p32.1	CN Loss	0.007	JUN
2p24.3	CN Loss	0.002	MYCN
2p23.1	CN Loss	0.002	ALK
2p16.1	CN Loss	0.002	REL
2q13	CN Loss	0.001	PAX8
2q31.1	CN Loss	0.001	HOXD13, HOXD11
5q13.1	CN Loss	0.002	PIK3R1
5q32	CN Loss	0.002	PDGFRB
5q35.1	CN Loss	0.002	NPM1
5q35.2	CN Loss	0.002	NSD1
6p21.33	CN Loss	0.006	POU5F1
7q31.2	CN Loss	0.002	MET
10q11.21	CN Loss	0.001	RET
10q23.31	CN Loss	0.001	PTEN
11p15.5	CN Loss	0.01	HRAS
11q13.3	CN Loss	0.004	CCND1
13q12.2	CN Loss	0.019	CDX2
13q14.2	CN Loss	0.019	RB1
15q24.1	CN Loss	0.001	PML
15q26.1	CN Loss	0.012	IDH2
16p13.3	CN Loss	0.002	TSC2
19p13.3	CN Loss	0.017	STK11
19p13.3	CN Loss	0.017	STK11, TCF3
19q13.2	CN Loss	0.012	AKT2
19q13.2	CN Loss	0.012	CD79A
20q13.32	CN Loss	0.019	GNAS
21q22.11	CN Loss	0.001	OLIG2
**High CNVs (CN gain)-BCR-ABL dependent and independent (TKIs-non-sensitive) group**			
Cytoband Location	Event	P-Value	CancerGeneCensus-Sanger.txt
1p36.13	CN Gain	0.002	SDHB
Cytoband Location	Event	P-Value	CancerGeneCensus-Sanger.txt
4p16.3	CN Gain	0.008	FGFR3, WHSC1
9q34.11 - q34.2	CN Gain	0.004	FNBP1, ABL1, NUP214, TSC1, RALGDS
17p13.3	CN Gain	0.001	YWHAE
17q12	CN Gain	0.004	CDK12
21q22.3	CN Gain	0.022	U2AF1

### Analysis of 13 CML samples with common transcriptomics and CNV

The 13 CML samples with common transcriptomics and CNV profiles were separately compared to control through TAC using the same filter criteria (p value = 0.001 and FDR value p value = 0.001). CNV-based clustering was similar to transcript-based clustering, except for samples CML 2, 4 and 10. These samples were in the same transcript cluster as undetectable-BCR-ABL transcript (Cluster-II-sub-cluster-II); in the CNV-based cluster, samples 2 and 4 were grouped in the higher CNV group (undetectable/<20% BCR-ABL transcript), and CML-10 was grouped in the CNV group with undetectable BCR-ABL transcript levels, new cases or un-detected BCR-ABL (Table 3).

**Table 3:** Transcriptionally clustered (at the significance level p=0.001 and FDR p=0.0001 and Fold Change<-4 or >4) 13-CML samples (Chronic Phase-new; CP-new and Blast new; B-new and Chronic Phase under treatment; CP-UT) which were also processed for CNVs-profiling

Cluster-I								
Sample-ID	CML-Phases and treatment status	Age/gender	Sample collection date	Time of Assessment	Treatment	Date of BCR-ABL assessment	BCR-ABL percentage	Comments based on BCR-ABL
CML-10	CP-New-10	20/M	28-05-2014	New	Imatinib	28-05-2014	120	Not known
						06-02-2015	0.95	
CML-02	CP-UT-2	50/M	22-05-2014	4 yrs	Initially with Droxygel (Antacid), Unidrea and later with Imatinib	14-02-2013	100%	Initially with Droxygel (Antacid), Unidrea and later with Imatinib-sensitive (TKI- sensitive)
						20-03-2014	0.12	
CML-04	CP-UT-4	14/M	22-05-2014	1.2 year	Imatinib	15-03-2013	100	Imatinib sensitive (TKI- sensitive)
						06-09-2014	0.95	
**Cluster-II**								
CML-13	CP-UT-13	50/M	29-05-2014	7.1 yrs	Droxygel (Antacid), Unidrea and Imatinib	20-02-2014	30	Droxygel (Antacid), Unidrea and Imatinib sensitive (TKI- sensitive)
						12-03-2015	0.1	
CML-21	CP-UT-21	60/M	05-06-2014	3.8 yrs	Droxygel (Antacid), Unidrea and Imatinib	14-11-2013	75	Droxygel (Antacid), Unidrea and Imatinib sensitive (TKI-sensitive)
						31-07-2014	0.11	
CML-36	CP-UT-36	23/M	03-07-2014	6.10 yrs	Initially Hydab and presently on Imatinib	20-02-2010	30	Initially Hydab and Imatinib sensitive (TKI- sensitive)
						30-10-2014	0.11	
CML-11	CP-UT-11	33/F	28-05-2014	4.2 yrs	Hydab and Imatinib	21-04-2010	97.54	Hydab and Imatinib (TKI-sensitive)
						21-05-2014	Not detectable	
**Cluster-III**								
CML-55	CP-UT-55	60/M	06-02-2015	3 months	Hydroxyurea, Zyloric and Imatinib	02-12-2014	98.47	Hydroxyurea, Zyloric and Imatinib non- sensitive
						01-05-2015	9.75	
CML-56	CP-UT-56	43/F	09-02-2015	1.0 yr	Hydroxyurea, Zyloric and Imatinib	29-02-2014	89.5	Hydroxyurea, Zyloric and Imatinib sensitive
						24-03-2015	1.2	
CML-58	CP-UT-58	32/F	09-02-2015	9 months	Hydroxyurea, Zyloric and Imatinib	12-04-2015	58.75	Hydroxyurea, Zyloric and Imatinib sensitive
						12-04-2015	9.8	
CML-57	CP-UT-57	42/F	09-02-2015	7 months	Hydroxyurea, Zyloric and Imatinib	22-07-2014	67.45	Hydroxyurea, Zyloric and Imatinib non-sensitive
						22-04-2015	8	
CML-59	CP-UT-59	15/M	01-04-2015	3 months	Hydroxyurea, Zyloric and Imatinib	11-12-2014	15.75	Hydroxyurea, Zyloric and Imatinib non-sensitive
						29-04-2015	9.75	
CML-14	B-New-14	35/F	29-05-2014	New	Hydroxyurea, Zyloric and Imatinib	09-06-2014	85.56	Not known

**Figure 2:**
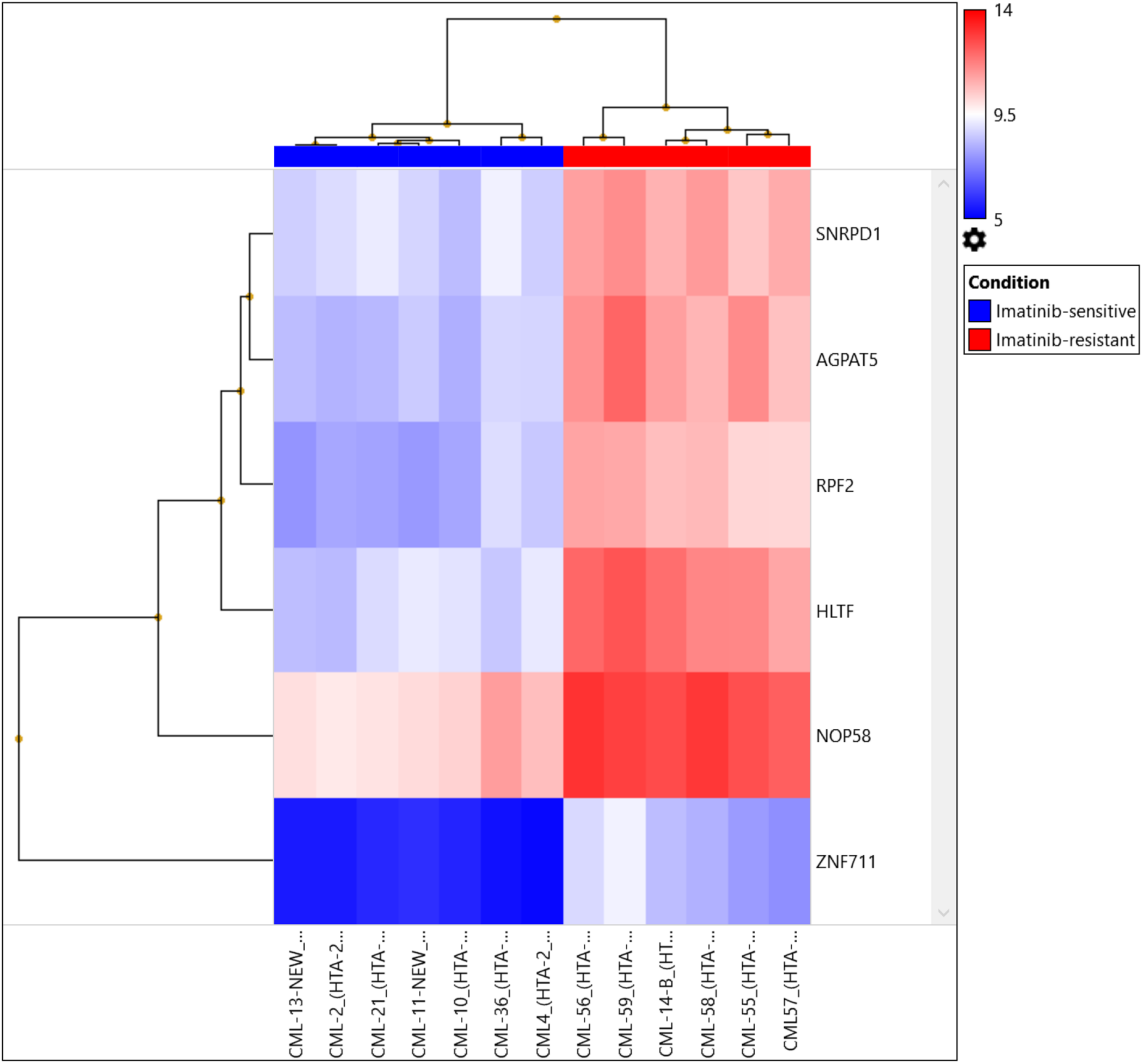
Hierarchical clustering of 13 CML samples (TKI-sensitive cases and TKI resistant cases) common between transcriptomics and CNV analysis were compared to control using the same filter criteria as for all CML cases and control at p=0.01

We identified up-regulation of 2,230 genes and down-regulation of 2,683 genes among Imatinib-resistant versus Imatinib-sensitive samples at the gene expression level after applying strict criteria [ANOVA p value < 0.001, FDR p value <0.0001, and gene fold-change < -4 or gene fold-change (linear) > 4, Figure 2, Table 3]. We identified highly significant up-regulation of helicase-like transcription factor (HLTF, p= 0.00000000589, FDR p= 0.0000492), small nuclear ribonucleoprotein D1 polypeptide (SNRPD1, p= 0.000000012, FDR p= 0.0000492), 1-acylglycerol-3-phosphate O-acyltransferase 5 (AGPAT5, p= 0.0000000191, FDR p= 0.0000633), NOP58 ribonucleoprotein (NOP58, p= 0.0000000347, FDR p= 0.0000711), ribosome production factor 2 homolog (RPF2, p= 0.0000000502, FDR p= 0.0000865) and zinc finger protein 711 (ZNF711, p= 0.000000058, FDR p= 0.0000933). The genes were up-regulated in the AGPAT5-mediated triacylglyceride (significance=2.27, p=0.005357), glycerophospholipid synthesis (significance=1.51, p=0.0031133), NOP58-mediated SUMOylation of RNA binding proteins (significance=1.89, p=0.012906), SNRPD1-mediated metabolism of non-coding RNA (significance=1.84, p=0.014454), mRNA processing (significance=1.55, p=0.028078), HLTF-mediated Retinoblastoma (RB) in cancer (significance=1.7, p= 0.019966), and E3 ubiquitin ligases ubiquitinate target proteins pathways (significance=1.63, p=0.023481).

**Figure 3:**
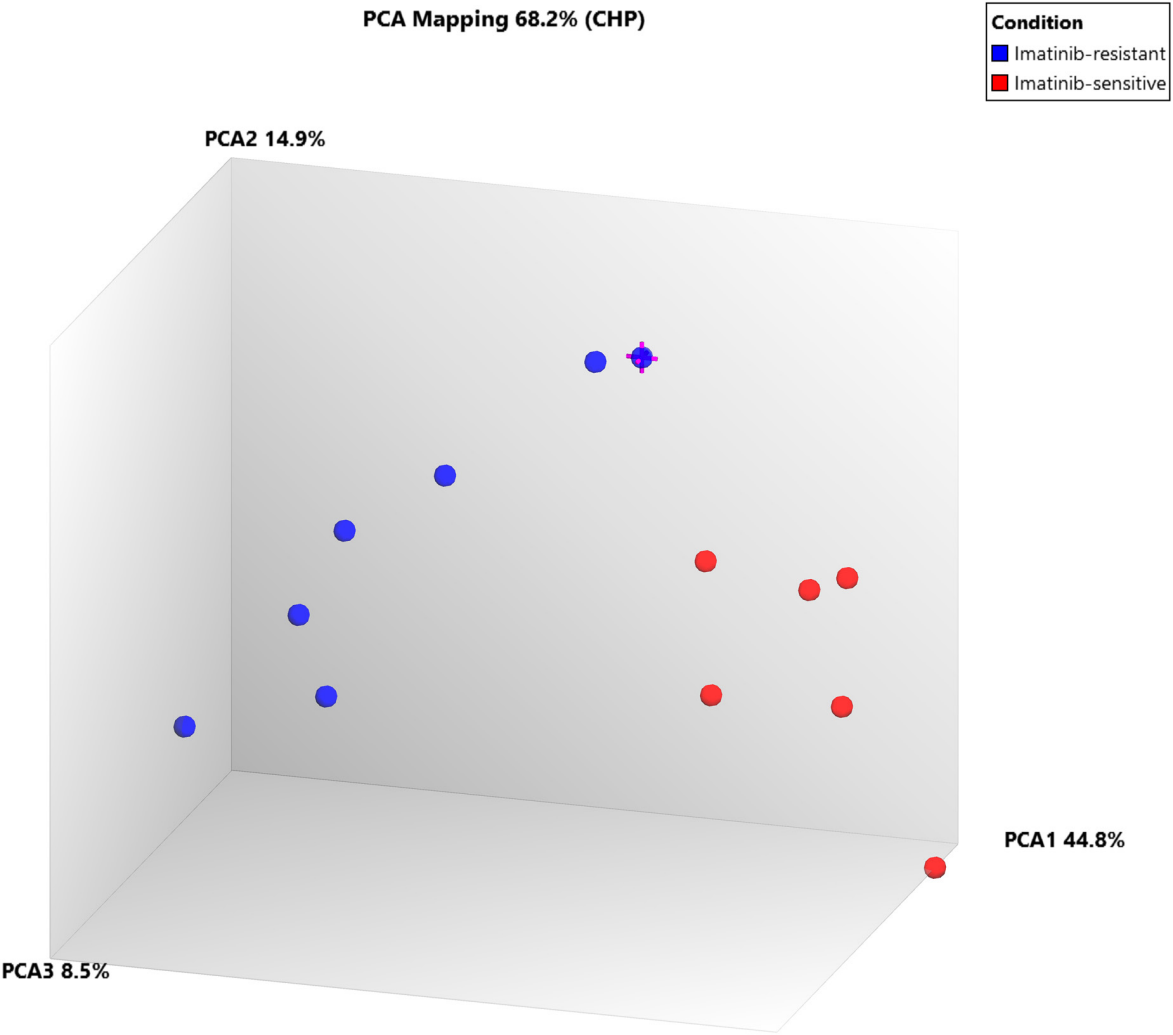
PCA plot between Cluster III (TKI-sensitive cases) and Cluster I+II (TKI resistant cases) at exonic-level using specific splicing index filter criteria [(1.) Exon Splicing Index (linear) < -4 or Exon Splicing Index (linear and exon expressed in atleast one condition) >4; 2.) ANOVA Exon p value < 0.001; Exon FDR p value <0.001. 3.) Gene fold change (linear and expressed in both conditions) < -5 or Gene fold change (linear) > 5], 0.01% coding-genes passed filter criteria

Further, when comparing Cluster III (TKI-resistant cases: CML-CP (CML-CP-UT 55-59, CML-B-14)) and Cluster I+Cluster-II (TKI sensitive cases: CML-CP-UT-2, 4, 10, 13, 21 and 36 and CML-CP-UT-11 as Imatinib/Imatinib plus other drugs-sensitive){ at the exon level using specific splicing index filter criteria [(1.) Exon Splicing Index (linear) < -4 or Exon Splicing Index (linear and exon expressed in at least one condition) >4; 2.) ANOVA exon p value < 0.001, exon FDR p value <0.001; 3.) gene fold-change (linear and expressed in both conditions) <-5 or Gene fold-change (linear) > 5], approximately 0.01% coding-genes passed filter criteria (Figures 2 and 3). Considering SI at the exon level, lysosomal protein transmembrane 4 beta (LAPTM4B, FC17.99), piezo-type mechanosensitive ion channel component 2 (PIEZO2, FC-8.36), angiopoietin 1 (ANGPT1, FC-6.04), complement factor H (CFH, FC-6.39), helicase-like transcription factor (HLTF, FC-8.44), serine palmitoyltransferase, long-chain base subunit 3 (SPTLC3, FC-5.23), 1-acylglycerol-3-phosphate O-acyltransferase 5 (AGPAT5, FC-7.30), CD109 molecule (CD109 FC-6.07), and zinc finger protein 711 (ZNF711, FC-6.91) were up-regulated in TKI-resistant cases. The up-regulation resulted from the following: a cassette exon splicing event in LAPTM4B (0.46), PIEZO2 (0.32), ANGPT1 (0.29), CFH (0.28), HLTF (0.28), and SPTLC3 (0.26); splicing of an alternative 3' acceptor site in AGPAT5 (0.22); and junctional splicing in CD109 and ZNF711. These genes were further processed using Reactome Pathway analysis software (https://reactome.org/), and hemostasis (Supplementary Figure 1) with specific involvement of Tie2 [11], Basigin-transmembrane glycoprotein signaling [12] (Supplementary Figures 2 and 3), CFH-mediated complement cascade, HTLF-mediated protein-ubiquitination, SPTLC3 mediated sphingolipid metabolism, and ZNF711-mediated RNA polymerase II transcription pathways were overexpressed in TKI-resistant cases.

### Validation of array-based transcripts by differential expression analysis

Down-regulated FOS, TGFβR2 and up-regulated TPX2 among all drug-treated CML cases as well as significantly up-regulated LAPTM4B, PIEZO2, ANGPT1, CFH, CD109 and HLTF molecule in TKI-resistant cases were validated in 23 Imatinib-treated CML cases. The samples were categorized on the basis of major molecular response (>1% and not-detected BCR-ABL copies). FOS and TGFβR2 down-regulated in ~48% of all Imatinib-treated CML cases (Table 4a and 4b). While TPX2 was up-regulated in 21.73% Imatinib-treated cases with >1% BCR-ABL copies and down-regulated or non-significant in most of the Imatinib-treated cases.

**Table 4:** Validation of (array-based) FOS, TGFβR2, TPX2, LAPTM4B, PIEZO2, ANGPT1, CFH, CD109 and HLTF transcripts by differential expression analysis in >1% and not detected %BCR-ABL 23 Imatinib-treated CML cases using beta actin and 18s ribosomal house-keeping gene as reference

	Duration	%BCR-ABL	Treatment	FOS	TGFBR2	TPX2	CFH	PIEZO2	CD109	ANGPTI	LAPTM4B	HLTF
**CML-1 (CP-UT)**	6 mths	5.56	Imatinib	NS	UP (≥0.020)		NS	UP (≥0.012)	NS	NS	UP (≥0.000)	UP (≥0.020)
**CML2 (CP-UT)**	4 yrs	38.78	Imatinib	Down (≥0.020)	UP (≥0.041)	Down (≥0.020)	Down (≥0.020)	Down (≥0.020)	Down (≥0.020)	Down (≥0.020)	NS	Down (≥0.020)
**CML-3 (CP-UT)**	6 mths	20.01	Imatinib	NS	NS	Down (≥0.040)	NS	UP (≥0.000)	NS	UP (≥0.030)	UP (≥0.000)	NS
**CML6 (CP-UT)**	3 yrs	4.37	Imatinib	Down (≥0.020)	NS	NS	Down (≥0.033)	NS	NS	Down (≥0.036)	NS	NS
**CML-7 (B-UT)**	6 mth	12.56	Imatinib	Down (≥0.031)	NS	NS	NS	UP (≥0.048)	NS	NS	UP (≥0.024)	NS
**CML-9 (CP-UT)**	5 yrs	4.32	Imatinib	NS	Down (≥0.000)	NS	NS	UP (≥0.000)	NS	UP (≥0.000)	UP (≥0.000)	NS
**CML12 (AP-UT)**	2 yrs	6.17	Imatinib	NS	NS	UP (≥0.020)	NS	NS	UP (≥0.020)	UP (≥0.041)	UP (≥0.011)	UP (≥0.000)
**CML13 (AP-UT)**	2 yrs	3.65	Imatinib	NS	Down (≥0.029)	NS	UP (≥0.000)	NS	UP (≥0.023)	UP (≥0.031)	UP (≥0.000)	NS
**CML14 (CP-UT)**	1 yrs	1.23	Imatinib	NS	Down (≥0.008)	NS	UP (≥0.031)	NS	UP (≥0.000)	UP (≥0.049)	UP (≥0.000)	NS
**CML-16 (CP-UT)**	3 mths	0.03	Imatinib	NS	Down (≥0.011)	NS	NS	UP (≥0.020)	NS	UP (≥0.000)	UP (≥0.032)	NS
**CML17 (CP-UT)**	3 mths	16.13	Imatinib	Down (≥0.036)	Down (≥0.036)	UP (≥0.000)	UP (≥0.000)	UP (≥0.000)	UP (≥0.034)	Down (≥0.036)	NS	UP (≥0.000)
**CML29 (B-UT)**	3 yrs	11.74	Imatinib	NS	Down (≥0.046)	NS	NS	NS	NS	NS	NS	NS
**CML31 (AP-UT)**	2 yrs	15.23	Imatinib	Down (≥0.039)	NS	UP (≥0.000)	UP (≥0.000)	UP (≥0.000)	UP (≥0.000)	UP (≥0.050)	NS	UP (≥0.000)
**CML32 (B-UT)**	2 yrs	6.85	Imatinib	Down (≥0.029)	NS	UP (≥0.000)	UP (≥0.000)	UP (≥0.000)	UP (≥0.000)	UP (≥0.000)	NS	UP (≥0.000)
**CML33 (B-UT)**	3 mths	126	Imatinib	Down (≥0.008)	Down (≥0.034)	NS	UP (≥0.030)	UP (≥0.028)	UP (≥0.033)	NS	NS	UP (≥0.008)
**CML37 (CP-UT)**	3 yrs	3.38	Imatinib	NS	NS	Down (≥0.042)	NS	NS	NS	NS	NS	UP (≥0.011)
**Percent -Up**				**6.666666**	**6.666667**	**26.66667**	**46.66667**	**60**	**53.33333**	**53.33333**	**53.33333**	**46.66666**
**Percent - Down**				**40**	**46.66667**	**20**	**13.33333**	**6.666667**	**6.666667**	**13.33333**	**0**	**6.666666**
**Sensitive Cases-**	**Duration**	**%BCR-ABL**	**Treatment**	**FOS**	**TGFBR2**	**TPX2**	**CFH**	**PIEZO2**	**CD109**	**ANGPTI**	**LAPTM4B**	**HLTF**
**CML8 (CP-UT)**	6 yrs	Not detected	Imatinib	NS	NS	NS	NS	NS	NS	NS	NS	UP (≥0.000)
**CML4 (CP-UT)**	5 yrs	Not detected	Imatinib	Down (≥0.038)	Down (≥0.038)	NS	Down (≥0.038)	NS	Down (≥0.038)	Down (≥0.049)	NS	Down (≥0.038)
**CML5 (CP-UT)**	2 yrs	Not detected	Imatinib	NS	NS	Down (≥0.031)	Down (≥0.031)	Down (≥0.020)	Down (≥0.031)	Down (≥0.042)	NS	Down (≥0.031)
**CML11 (CP-UT)**	3 yrs	Not detected	Imatinib	Down (≥0.047)	Down (≥0.047)	Down (≥0.047)	Down (≥0.047)	NS	Down (≥0.047)	Down (≥0.038)	NS	Down (≥0.047)
**CML34 (CP-UT)**	4 yrs	Not detected	Imatinib	Down (≥0.036)	Down (≥0.036)	Down (≥0.036)	NS	NS	NS	NS	NS	Down (≥0.036)
**CML10 (CP-UT)**	2 yrs	Not detected	Imatinib	Down (≥0.024)	Down (≥0.019)	NS	NS	NS	Down (≥0.021)	Down (≥0.041)	NS	NS
**CML15 (CP-UT)**	3 yrs	Not detected	Imatinib	NS	NS	NS	NS	Down (≥0.038)	NS	NS	NS	NS
**Percent -Up**				**0**	**0**	**0**	**0**	**0**	**0**	**0**	**0**	**14.28571**
**Percent - Down**				**57.14285714**	**57.14286**	**42.85714**	**14.28571**	**6.666667**	**57.14286**	**57.14286**	**0**	**57.14285**

NS = Non-sensitive.

LAPTM4B (53.33% cases), PIEZO2 (60% cases), ANGPT1 (53.33% cases), CFH (46.66% cases), CD109 (53.33% cases) and HLTF (46.66% cases) molecule were up-regulated in >1% BCR-ABL copies Imatinib-treated CML cases and 57.14%, 14.28%, 57.14%, 6.66%, 0%, 57.14%, HLTF, CFH, CD109, PIEZO2, LAPTM4B and ANGPT1 respectively down-regulated in not detected BCR-ABL copies (Table 4a and 4b).

### Biobank genotyping of CML resistant cases

Axiom Biobank genotyping data was analyzed through automated Genotyping Console Software, which includes allele-calling algorithms and user-friendly visualization tools. All analyzed samples passed QC, and 99.571% was the average call rate. An explanation of the SNP metrics summary is provided in Supplementary Table 4. On the basis of gender, 69 samples were from male patients, and 27 samples were from female patients.

pLink software (http://zzz.bwh.harvard.edu/plink/) was used to perform a range of basic, large-scale analyses in a computationally efficient manner. Associations between individual SNPs and CML risk were assessed using p=0.001 and ORs > 4.0 and 95% CIs derived from logistic regression models.

**Figure 4:**
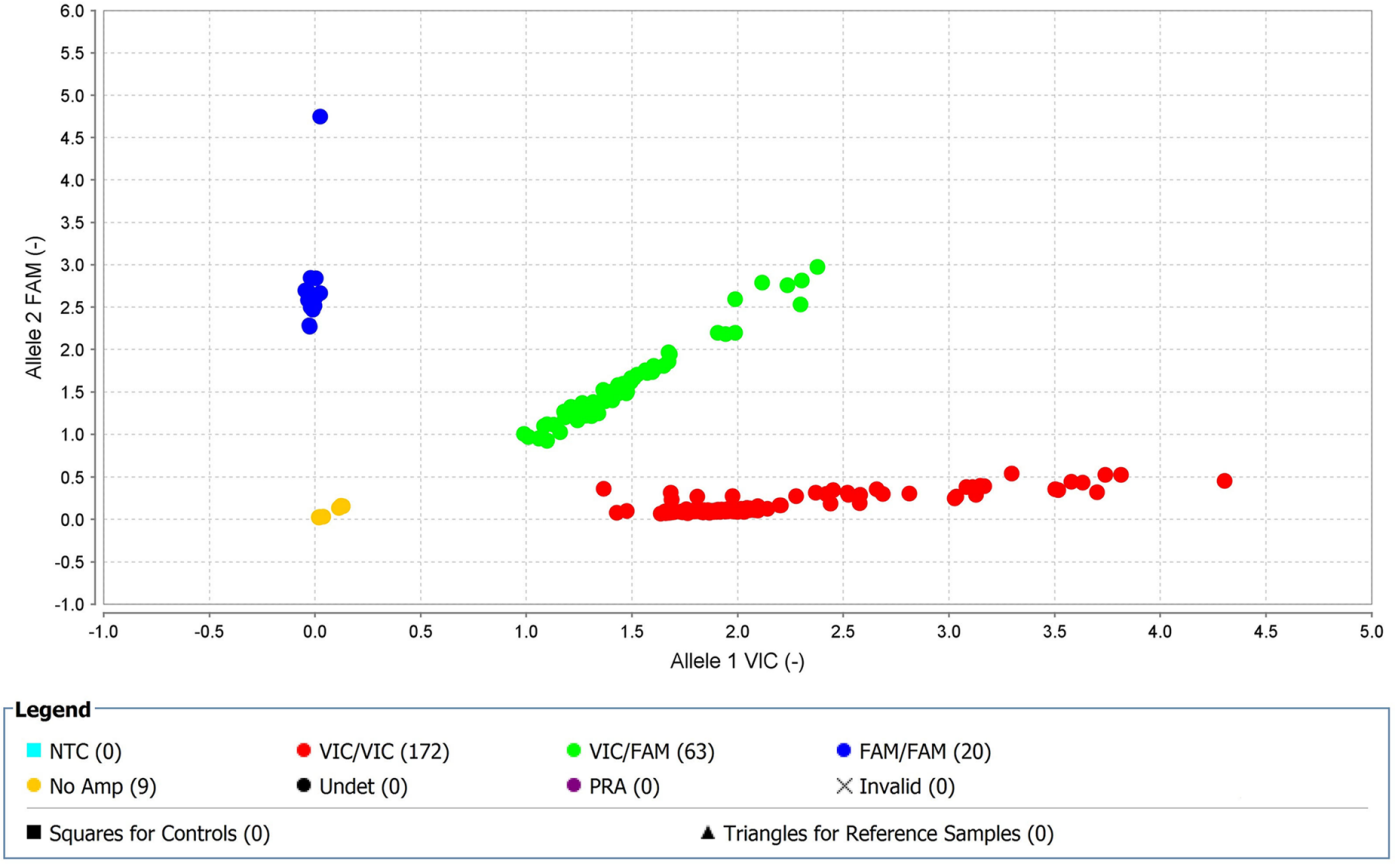
With high-quality threshold the each sample is clustered on the basis of genotype call Here the cluster observed shows the presence of FAM-labelled allele-2 (C) in all samples in rs239798. The yellow spots are the negative controls.

**Figure 5:**
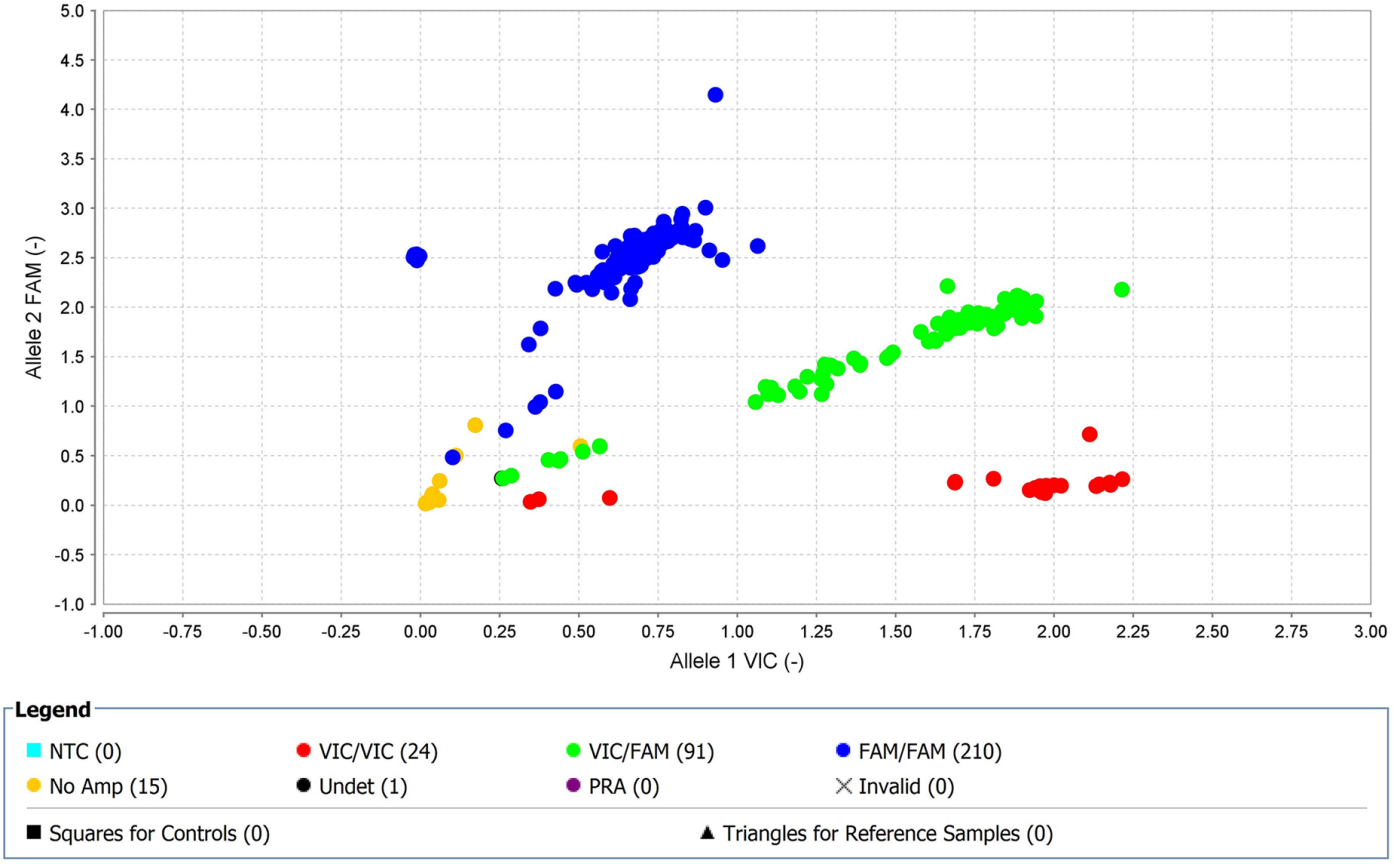
With high-quality threshold the each sample is clustered on the basis of genotype call Here the cluster observed shows the presence of FAM-labelled allele-2 (A) in all samples in rs9475077. The yellow spots are the negative controls.

Seventeen SNPs reached genome-wide significance (p=0.001) for TKI-treated CML samples (2 SNPs on chromosome 1, 2 SNPs on chromosome 2, 1 SNP on chromosome 4, 1 SNP on chromosome 5, 4 SNPs on chromosome 6, 1 SNP on chromosome 12, 2 SNPs on chromosome 13, 2 SNPs on chromosome 16, 1 SNP on chromosome 20, and 1 SNP on chromosome 21) (Supplementary Table 5a). Furthermore, regional LD plot was generated for each query SNP (identified through pLink software) through SNAP Proxy search software using r2 threshold=0.8, a distance limit between query and proxy SNP=500, 1000 genomes pilot 1 data-set from the 1000 Genomes Project, which uses phased genotypes for 179 individuals from the HapMap CEU (Utah residents with Northern and Western European ancestry from the CEPH collection), YRI (Yoruba in Ibadan, Nigeria), and JPT+CHB (combined panel of Japanese in Tokyo, Japan and Han Chinese in Beijing, China) panels. From these analyses, rs239798 showed complete correlation with rs9475077, such that r2=1 at a maximum distance of 801 (Supplementary Table 5b). rs12057639 was correlated with rs1327107 (r2=0.934 at a distance of 1, 03, 649) (Supplementary Table 5b). Association plots for rs9475077 and rs12057639 are shown in Supplementary Figure 4a and 4b. Importantly, both rsID239798 (Ch6:54940890) and rsID9475077 (Ch6:54941691) were associated with FAM83B. Hence, we validated rs239798 and rs9475077 with the Taqman genotyping protocol (Figures 4 and 5, Table 5) and identified similar allele frequency as observed through Axiom Biobank Array.

**Table 5:** Genotyping of rs2854344 and rs9475077 through unlabeled PCR primers and TaqMan® MGBprobes (FAM ™ and VIC® dye-labeled in 40X assay mix

rs239798							
Population	Allele 1 Frequency	Allele 2 Frequency (Minor Allele Frequency-C)	1/1 Frequency	1/2 Frequency	2/2 Frequency	Chi-Squared	P-Value
CML	0.728395062	0.27160494	0.5617284	0.333333333	0.10493827	4.021168	0.044937
Control	0.933333333	0.06666667	0.9	0.066666667	0.03333333	19.40051	0
**rs9475077**							
**Population**	**Allele 1 Frequency (Minor Allele Frequency-A)**	**Allele 2 Frequency**	**1/1 Frequency**	**1/2 Frequency**	**2/2 Frequency**	**Chi-Squared**	**P-Value**
CML	0.282978723	0.71702128	0.1021277	0.361702128	0.53617021	2.775499	0.045747
Control	0.033333333	0.96666667	0	0.066666667	0.93333333	0.107015	0.743586

## DISCUSSION

This study provides evidence to support that, in addition to the BCR-ABL translocation t(9;22) (q34;q11), specific gene abnormalities contribute to the transformation from CML-chronic phase (CML-CP) with no copy number aberrations (CNAs) to CML-blast crisis (CML-BC) in adult and pediatric CML [13–17]. In pediatric CML-BC of lymphoid origin, deletions in IKZF1, PAX5, and/or CDKN2A have been frequently reported [13, 14]. In adults, Hosoya *et al.* performed genome-wide screening of DNA in a total of 55 CML patients at different stages using a high-resolution array-based comparative genomic hybridization technique [18]. They identified losses in 2q26.2-q37.3, 5q23.1-q23.3, 5q31.2-q32, 7p21.3-p11.2, 7q31.1-q31.33, 8pter-p12(p11.2), 9p, and 22q13.1-q13.31 and gains in 3q26.2-q29, 6p22.3, 7p15.2-p14.3, 8p12, 8p21.3, 8p23.2, 8q24.13-q24.21, 9q, 19p13.2-p12, and 22q13.1-q13.32 in chronic phase and reported that these alterations occurred at a higher frequency in AP and blast crisis [18]. Another study by Brazma *et al.* [19] reported losses at 1p36, 5q21, and 9p21 and gains at 1q, 8q24, 9q34, 16p, and 22q11 after genome-wide screening at a resolution of 1 Mb among 54 samples at different stages of CML together with 12 CML cell lines. Furthermore, Mullighan *et al.* [20] found higher CNAs in CML-AP and CML-BC of lymphoid origin (1.14 and 7.8, respectively) compared to CML-CP (0.47) using SNP array analysis of 34 adult CML cases [20].

Compared to the references described above [18–20] in undetectable-BCR-ABL-TKI-sensitive group, we similarly observed the previously reported loss of 7p15.2-HOXA9, HOXA11, and HOXA13. In BCR-ABL-dependent/independent TKI-non-sensitive group, we also observed the previously reported loss of 1p36 (TNFRSF14, PRDM16), 2q31.1 (HOXD13, HOXD11), 5q32(PDGFRB), and 7q31.2 (MET) and gain of 9q34.11-q34.2 (FNBP1, ABL1, NUP214, TSC1, and RALGDS genes (Table 2b and 2c). Hence, through above references and our study, we conclude that CNAs were absent in CML-CP-New and CML-CP-UT-TKI-sensitive cases. However, these reported variations were relatively common in samples at progressed stages and TKI-non-sensitive cases. These observations support the notion that the BCR-ABL fusion protein is sufficient to induce CML, but additional genomic changes are required for disease progression and play important roles in resistance to TKI [13–17].

Further, genome-wide transcriptomics have also provided insight into the mechanisms of distinction between CP and BC, progression and resistance of CML on the whole blood of different phases of CML-patients, cell lines, leukemia stem cells, and normal stem and progenitor cell populations [6, 10, 17] Radich *et al*. [6] showed an association of decreased expression of Jun B and Fos with other deregulated pathways with early accelerated phase and identified 6 genes (NOB1, DDX47, IGSF2, LTB4R, SCARB1, and SLC25A3) that discriminated CP from BC [6]. Later, Wang *et al.* [17] reported over-expression of early erythroid-related factors [9, 21] transcription factors and activation of proliferative markers like ERK/MAPK, JAK-STAT, and ErbB pathways in K562 cell line [22]. Gerber *et al.* [10] performed genome-wide transcriptome analysis of CML leukemia stem cells and normal stem and progenitor cell populations using exon arrays. They identified 97 genes that were differentially expressed in CML versus normal stem and progenitor cells. These included significantly up-regulated cell surface genes and genes involved in oxidative metabolism, DNA repair pathways and the activation of inflammatory cytokines. They also observed down-regulation of pro-differentiation and TGF-β/BMP signaling pathways [10]. However, methylation and down-regulation of 897 genes including tumor-suppressor genes or regulators of cell proliferation were observed during disease progression, i.e., conversion of CP to AP/Blast [23].

We also identified up-regulation of highly significant proliferative (24 genes in the RB pathway, ARG1 and CDK1), cell cycle (6 genes involved in G1 to S cell cycle control and 12 genes involved in Mitotic G1-G1/S phases), replicative (8 genes involved in DNA replication) and DNA repair markers (9 genes involved in DNA repair) and down-regulation of several genes related to the immune system (10 genes involved in allograft rejection and 12 genes involved in the Vitamin D receptor pathway), TCR signaling, TGF-beta signaling (FOS, FOSB, TGFBR2, ETS1, JUNB, and LIMK2) and chemokine signaling pathway (CCR6, CCR4, CCR7, CX3CR1, XCL1, CXCL16, JAK3, LYN, ITK, and TIAM1) (Supplementary Table 2) in all drug-treated CML samples when compared against control.

At the exon level, high splicing index affected the dysregulation of normal cellular processes in drug-treated CML cases, including up-regulation of Myeloperoxidase (MPO)-induced neutrophil degranulation affecting the innate immune system [24], TPX2 up-regulation, suggesting inhibition of TP53 transcriptional activity [25, 26], and TYMS, suggesting CML cells are more within the G1/S transition through more formation of TMP and dihydrofolate. Overall, these three factors affected the up-regulation of cell cycle (Reactome Analysis). Further, down-regulation of SKAP1 (Src Kinase Associated Phosphoprotein 1), ITK and TGFBR2 and FOS in CML cases led to down-regulation of the TCR signaling and immune system pathways Additionally, the Src-family kinases (SFKs) have been implicated in BCR-ABL signaling and in the progression of CML [27]. ITK and FOS are involved in the down-regulation of TCR signaling and immune system, respectively, as reported by [6, 10]. On validation in more samples, FOS and TGFBR2 were down-regulated in ~ fifty percent cases and were independent of major molecular response. Contrarily, TPX2 was up-regulated in cases with >1% BCR-ABL copies further validate suppression of TP53 transcriptional activity in resistance [25, 26].

Further, CML-resistant versus -sensitive cases at the exon level, due to high splicing index, nine genes were differentially expressed. Out of which six genes were further validated in additional resistant (>1% BCR-ABL copies in CML-CP-UT, CML-AP-UT, CML-B-UT,) and sensitive (undetectable levels BCR-ABL copies in CML-CP-UT) cases. These genes showed specific involvement of Tie2 (Supplementary Figures 1 and 2) and Basigin-transmembrane glycoprotein (Supplementary Figure 3) in Imatinib-resistant CML cases as reported previously [11, 12]. Tie-2 receptor tyrosine kinase and its ligand ANGPT1 are involved in CML progression or resistance [28]. Basigin also plays important role in tumor invasion, as it is co-expressed in the presence of high lactate and has been reported as a poor prognostic indicator in GIST [35] and imatinib-resistant indicator in CML cells [29]. Amplification of LAPTM4B, which contributes to chemotherapy resistance and recurrence of breast cancer [30, 31] and other solid tumors [32] was over-expressed in our TKI-resistant CML cases. Additionally, inactive EGFR complexes with LAPTM4B recruits Sec5exocyst sub complex which binds to autophagy inhibitor and activates autophagy [33]. Up-regulation of HLTF is associated with tumor progression in hypopharyngeal and cervical cancers [34, 35] was over-expressed in our TKI-resistant CML cases. Recently, Cipolla *et al* [36] suggested that HLTF repairs DNA damage by acting as a ubiquitin ligase caused by drug-induced reactive oxygen species, leading to resistance [36] and also modulates lysosomal autophagy [37]. CD109 regulates TGF-β receptor endocytosis and degradation to inhibit TGF-β signaling [38] and over-expression of CD109 in Imatinib resistant cases may further down-regulate TGF- β signaling. CFH co-factor for complement factor 1 inhibits C3 activation cascade in alternative pathway by promoting cleavage of C3b to iC3b-over-expression has been reported in cutaneous squamous cell carcinoma cells [39]. Expression of CFH help in immune escape and it has been documented in malignant ovarian and bladder cancers [40]. CFH also controls the stemness of liver cancer cells [41]. PIEZO2 which has been proposed as biomarker for CML stem cells [8] was also over-expressed in our Imatinib resistant cases.

Analysis of CML patients in the chronic phase and under treatment (CML-CP-UT) with BCR-ABL10-77.02% as identified through the genotyping array, which has been designed for a broad range of applications to give us complete flexibility both for identification of genotypic markers and to explore the complexity of diseases [42, 43], we found that rsID239798 (Ch6:54940890) and rsID9475077 (Ch6:54941691) were associated with FAM83B. FAM83B is a proto-oncogene involved in the epidermal growth factor receptor (EGFR) signaling pathway and activates both the EGFR itself and downstream RAS/MAPK and PI3K/AKT/TOR signaling cascades [44–46]. Both the rsIDs are missense mutations and show a minor allele frequency of C=0.2895/1450 (rsID239798; lysine (K) to Threonine (T) transition at position 640) and A=0.2893/1449 (rsID9475077; threonine (T) to Asparagine (N) transition at position 907) as established by 1000 Genome Project [47]. Grant [48] also suggested a role for FAM83A and FAM83B in therapeutic resistance to TKI [49].

## MATERIALS AND METHODS

### Ethics statement

All the samples were obtained after informed consent, and the study was approved by the Institutional Ethics Committee, King George's Medical University. All experiments have been performed in accordance with relevant guidelines and regulations.

### Patients and sample preparation

We collected peripheral blood samples from 4 healthy volunteers and 70 clinically diagnosed CML patients, which included treated and new chronic phase (CML-CP-UT and CML-CP-New), treated CML-accelerated phase (CML-AP-UT), and treated fresh blast phase (CML-B-UT and CML-B-New) samples from the Department of Clinical Hematology, King George's Medical University, Lucknow, India. All CML samples displayed a myeloid phenotype. More than 90% of patient cells were Philadelphia chromosome-positive (Ph), and blast crisis was characterized by >30% or >50% blasts and promyelocytes in peripheral blood or bone marrow, respectively [50].

All patients (Table 6) were treated with Imatinib alone or in combination with Hydrea/Hydroxyurea. Importantly, when patients failed to achieve time-dependent molecular targets, we switched to nilotinib or high-dose Imatinib. Clinical outcomes included molecular response based on BCR-ABL. Additionally, because patients were from a remote area, the samples used in the study were collected on dates other than the date of disease initiation or the date on which BCR-ABL expression was assessed.

**Table 6:** Chronic myeloid leukemia - clinical presentation showing different phases without (Chronic Phasenew; CP-new and Blast new; B-new) and with (Chronic Phase under treatment; CP-UT, accelerated phase under treatment; AP-UT and Blast Phase under treatment; B-UT ) treatment for the samples being processed for copy number variations and transcriptomics analysis

Chronic myeloid Leukemia-Chronic Phase New (CML-CP-New)										
	Sample ID	Age/Sex	Sample collection date	Time of Assessment	Date disease initiated	Follow up date	Treatment	Date of BCR-ABL detection	BCR-ABL%	Comments based on BCR-ABL
1	9	46/M	28-05-2014	New	28-05-2014	28-05-2015	Imatinib	28-05-2014	100	-
2	10	20/M	28-05-2014	New	28-05-2014	30-03-2015	Imatinib	28-05-2014	120	-
								06-02-2015	0.95	-
3	30	22/M	12-06-2014	New	24-06-2014	24-06-2014	Imatinib	24-06-2014	100	-
								30-09-2014	12.65	
4	73	50/M	22-04-2015	New	25-04-2015	29-04-2015	Imatinib	25-04-2015	73.8	-
5	83	70/M	27-04-2015	New	18-04-2015	01-05-2015	Imatinib	18-04-2015	77.02	-
**Chronic myeloid Leukemia-Chronic Phase Under Treatment (CML-CP-UT)**										
	**Sample No**	**Age/Sex**	**Sample collection date**	**Time of Assessment**	**Date disease initiated**	**Follow up date**	**Treatment**	**Date of BCR-ABL detection**	**BCR-ABL%**	**Comments based on BCR-ABL**
6	2	50/M	22-05-2014	4 yrs	10-06-2010	20-03-2014	Droxygel (Antacid), Unidrea and Imatinib	14-02-2013	100%	Initially with Droxygel (Antacid), Unidrea and later with Imatinib sensitive
								20-03-2014	not detected	
7	4	14/M	22-05-2014	10 months	04-07-2013	20-04-2015	Imatinib	15-07-2013	100	Imatinib sensitive
								06-09-2014	Not detected	
								18-03-2015	81.88	
8	6	24/M	22-05-2014	7 months	21-11-2013	22-06-2015	Imatinib	21-11-2013	75	Imatinib sensitive
								17-06-2014	0.09	
9	7	41/M	22-05-2014	7.2 yrs	29-03-2007	21-05-2015	Imatinib	28-02-2007	100%	Imatinib sensitive
								22-07-2014	not Detected	
10	11	33/F	28-05-2014	4.2 yrs	21-05-2014	23-04-2015	Hydab and Imatinib	21-04-2010	97.54	
								21-05-2014	not Detected	Hydab and Imatinib sensitive
11	13	50/M	29-05-2014	7.1 yrs	26-04-2007	06-07-2015	Droxygel (Antacid), Unidrea and Imatinib	20-02-2014	30	Droxygel (Antacid), Unidrea and Imatinib sensitive
								12-03-2015	0.1	
12	16	30/M	29-05-2014	10 months	23-03-2015	19-03-2015	Imatinib	23-05-2014	43.2	Imatinib non-sensitive
								19-03-2015	35	
13	20	33/F	05-06-2014	1 month	21-05-2014	05-06-2014	Imatinib	21-05-2014	97.54	NA
14	21	60/M	05-06-2014	3.8 yrs	09-09-2010	11-06-2015	Droxygel (Antacid), Unidrea and Imatinib	14-11-2013	75	Droxygel (Antacid), Unidrea and Imatinib-sensitive
								31-07-2014	0.11	
15	23	40/M	05-06-2014	2.4 yrs	24-02-2012	05-06-2014	Unidrea and Imatinib, Nilotinib	31-07-2013	28.35	Unidrea and Imatinib, Nilotinib-non-sensitive (TKI non sensitive)
								13-05-2014	11.9	
								16-02-2015	16.53	
16	24	26/F	05-06-2014	8.5 yrs	05-01-2006	21-05-2015	Initially treated with Myeleron, Hydab irocos, Zyloric since may 2005 on Imatinib	24-04-2014	30	Initially treated with Myeleron, Hydab irocos, Zyloric since may 2005 on Imatinib non-sensitive
								21-05-2015	15.85	
17	26	32/M	05-06-2014	2.1 yrs	10-05-2012	22-09-2014	Imatinib	26-03-2012	100	Imatinib non-sensitive
								10-05-2014	13.6	
18	36	23/M	03-07-2014	6.10 yrs	08-09-2007	07-05-2015	Hydab and Imatinib	20-02-2010	30	Hydab and Imatinib-sensitive
								30-10-2014	not detected	
19	43	27/M	10-07-2014	7 yrs	21-07-2007		Initially Hydroxyurea and Imatinib	23-02-2012	55.89	Imatinib non-sensitive
								17-06-2014	11.18	
								14-03-2015	0	
20	48	33/M	31-07-2014	10.4 yrs	16-03-2004	18-09-2014	Initially Hydroxyurea and since December 2004 Imatinib	01-10-2004	100	Imatinib non-sensitive
								07-03-2013	60	
								05-09-2013	41.1	
								03-09-2013	41.41	
								29-05-2014	9.94	
21	50	21/M	31-07-2014	4.3 yrs	01-04-2010	18-09-2014	Imatinib	20-11-2013	23.3	Imatinib non-sensitive
								13-08-2014	10.12	
22	51	60/M	31-07-2014	25 year			Initially treated with Myeleron, Hydab irocos, Zyloric since may 2005 on Imatinib	12-05-2013	30	Initially treated with Myeleron, Hydab irocos, Zyloric since may 2005 on Imatinib non-sensitive
								11-06-2014	8.98	
23	52	40/M	31-07-2014	4.3 yrs	01-04-2010	31-07-2014	Imatinib	10-04-2010	100	Imatinib non-sensitive
								01-07-2014	18	
24	53	24/M	08-01-2015	3.1 yrs	08-12-2011	08-01-2015	Imatinib	08-12-2011	100	Imatinib non-sensitive
								22-02-2015	17.23	
25	54	28/M	06-02-2015	4.4 yrs	15-10-2010	22-12-2014	Imatinib	15-05-2014	100	Imatinib non-sensitive
								06-11-2014	40.4	
26	55	60M	06-02-2015	3 months	02-12-2014	01-05-2015	Hydroxyurea, Zyloric and Imatinib	02-12-2014	98.47	Hydroxyurea, Zyloric and Imatinib-non- sensitive
								01-05-2015	9.75	
27	56	43F	09-02-2015	1.0 yr	29-02-2014	24-03-2015	Hydroxyurea, Zyloric and Imatinib	29-02-2014	89.5	Hydroxyurea, Zyloric and Imatinib non- sensitive
								24-03-2015	1.2	
28	57	42F	09-02-2015	7 months	22-07-2014	22-04-2015	Hydroxyurea, Zyloric and Imatinib	22-07-2014	67.45	Hydroxyurea, Zyloric and Imatinib non- sensitive
								22-04-2015	8	
29	58	32/F	09-02-2015	9 months	12-04-2015	12-04-2015	Hydroxyurea, Zyloric and Imatinib	12-04-2015	58.75	Hydroxyurea, Zyloric and Imatinib non- sensitive
								12-04-2015	9.8	
30	59	15/M	01-04-2015	3 months	10-12-2014	13-04-2015	Hydroxyurea, Zyloric and Imatinib	11-12-2014	15.75	Hydroxyurea, Zyloric and Imatinib non- sensitive
								29-04-2015	9.75	
31	60	29/F	09-04-2015	4.5 yrs	28-12-2010		Imatinib	08-04-2011	18	Imatinib non-sensitive
								24-02-2015	16	
32	61	26/F	09-04-2015	9.4 yrs	18-12-2005		Imatinib	08-04-2011	18%	Imatinib non-sensitive
								24-02-2014	BCR-ABL positive in 200 cells	
33	63	37/M	09-04-2015	7 months	21-08-2014	09-04-2015	Imatinib	21-08-2014	100	Imatinib non-sensitive
								09-04-2015	80	
34	65	43/M	20-04-2015	7.9 yrs	29-06-2007	22-04-2015	Imatinib	24-06-2014	18.95	Imatinib non-sensitive, however, nilotinib sensitive
								22-04-2015	4.63	
35	66	30/F	20-04-2015	4.4yrs	15-05-2010	22-12-2014	Imatinib	15-05-2014	100	Imatinib non-sensitive
								06-01-2015	40.4	
36	67	52/M	16-04-2015	6.1 yrs	21-02-2009	01-06-2015	Initially Hydab, Uridrea and since october 2012 Imatinib	15-04-2014	20	Initially Hydab, Uridrea and since october 2012 Imatinib-non-sensitive
								01-06-2015	18.63	
37	68	28/M	16-04-2015	4 months	01-11-2014	08-06-2015	Imatinib	22-09-2014	45.2	Imatinib non-sensitive
								22-04-2015	16.1	
38	71	52/M	16-04-2015	8.1 yrs	21-02-2007	21-06-2015	Initially Hydab, Uridrea and since october 2012 Imatinib	15-04-2014	20	Initially Hydab, Uridrea and since october 2012 Imatinib non-sensitive
								22-06-2015	8.63	
39	75	20/M	27-04-2015	2.1 yrs	02-03-2013	02-05-2015	Initially Hydab, Uridrea and since march 2013 Imatinib	02-03-2013	100	Initially Hydab, Uridrea and since march 2013 Imatinib non-sensitive
								01-04-2015	20	
40	77	25/M	27-04-2015	7.2 yrs	19-02-2008	03-05-2015	Imatinib	19-02-2008	80	Imatinib non-sensitive
								03-04-2015	8	
41	78	30/F	27-04-2015	3.1 yrs	21-03-2013	03-05-2015	Imatinib	28-10-2013	74.91	Imatinib non-sensitive
									10	
42	79	45/M	27-04-2015	2.3 yrs	17-01-2013	27-04-2015	Imatinib	10-01-2013	110	Imatinib non-sensitive
								04-03-2015	39.09	
43	80	26/M	27-04-2015	3.2 yrs	10-02-2012	03-05-2015	Imatinib	10-02-2012	80	Imatinib non-sensitive
								03-04-2015	29	
44	81	26/F	27-04-2015	2.6 yrs	25-10-2012	03-05-2015	Imatinib	25-10-2012	100	Imatinib non-sensitive
								03-04-2015	31.02	
45	84	23/M	27-04-2015	8 yrs	26-04-2007	27-04-2015	Imatinib	10-12-2014	20	
								27-03-2015	8	
46	85	30/M	27-04-2015	9.7yrs	13-09-2005	03-05-2015	Hydab and Imatinib	08-07-2005	110	Hydab and Imatinib non-sensitive
								02-02-2015	13.12	
47	87	45/F	08-06-2015	2.9 yrs	14-09-2011	08-06-2015	Imatinib	01-07-2015	86	Imatinib non-sensitive
48	88	48/M	08-06-2015	4.8 yrs	28-10-2010	08-06-2015	Imatinib	17-07-2014	30	Imatinib non-sensitive
								13-05-2015	10	
49	89	25/M	08-05-2015	8 months	23-08-2014	08-06-2015	Imatinib	23-08-2014	100	Imatinib non-sensitive
								24-03-2015	2	
50	91	66/F	08-06-2015	6 months	10-11-2014	08-06-2015	Imatinib	10-09-2014	38.52	Imatinib non-sensitive
								22-04-2015	26.1	
51	93	60/M	08-06-2015	11 months	17-07-2014	08-06-2015	Imatinib	14-05-2014	100	Imatinib non-sensitive
								11-03-2015	70.13	
**Chronic Myeloid Leukemia-Accelerated Phase-Under Treatment (CML-AP-UT)**										
	**Sample No**	**Age/Sex**	**Sample collection date**	**Time of Assessment**	**Date disease initiated**	**Follow up date**	**Treatment**	**Date of BCR-ABL detection**	**BCR-ABL%**	**Comments based on BCR-ABL**
52	1	35/M	22-05-2014	1.8 yrs	20-09-2012	30-03-2015	Imatinib	30-08-2012	26	Imatinib non-sensitive
53	22	40/M	05-06-2014	5.2 yrs	02-04-2009	18-06-2014	Hydab and Imatinib	17-11-2013	39.07	Hydab and Imatinib non-sensitive
								05-08-2014	20.11	
54	32	27/F	12-06-2014	15 yrs	02-06-1999	30-03-2015	Initially treated with Myeleron, Hydab irocos, Zyloric since may 2005 on Imatinib	13-07-2014	55.63	Initially treated with Myeleron, Hydab irocos, Zyloric since may 2005 on Imatinib-non-sensitive
								17-11-2014	35.37	
								30-03-2015	26.93	
55	33	20/M	28-06-2014	2 yrs	19-07-2012	18-06-2015	Imatinib	24-07-2012		Imatinib non-sensitive
								27-06-2014	12.18	
								14-03-2015	0	
56	35	28/F	03-07-2014	6.3 yrs	03-04-2008	03-07-2014	Imatinib	24-07-2010	100	Imatinib non-sensitive
								13-04-2013	0.02	
								03-07-2014	8.56	
57	37	27/F	03-07-2014	8 months	19-12-2013	04-06-2015	Imatinib	01-04-2013	100	Imatinib non-sensitive
								03-07-2014	50	
58	38	60/M	03-07-2014	1.5 yrs	21-02-2013	13-03-2014	Imatinib	29-03-2013	79.01	Imatinib non-sensitive
								24-06-2014	35.12	
59	62	42/M	09-04-2015	4.4 yrs	04-11-2010	09-04-2015	Uridrea, Imatinib and since February 2014 Nilotinib	06-06-2014	14.96	Uridrea, Imatinib was non-sensitive but Nilotinib was TKI-non-sensitive
								22-06-2015	4.03	
60	69	40/F	16-04-2015	3 months	29-12-2014	13-04-2015	Imatinib	04-11-2014	14.08	Imatinib non-sensitive
								29-04-2015	9.75	
61	70	30/M	16-04-2015	1.0 year	01-04-2014	22-04-2015	Imatinib and december 2014 nilotinib	10-01-2015	20	Imatinib and Nilotinib sensitive
								22-04-2015	1.63	
62	72	45/M	16-04-2015	6 months	29-10-2014	08-06-2015	Imatinib and june 2015 Nilotinib	16-10-2014	25.19	Imatinib non-sensitive
								01-05-2015	5.45	
63	86	49/M	27-04-2015	5 months	11-12-2014	08-06-2015	Imatinib and march 2015 Nilotinib	25-11-2014	18.04	Imatinib non-sensitive
								01-05-2015	2.45	
**Chronic Myeloid Leukemia-Blast Phase New (CML-BP-New)**										
	**Sample No**	**Age/Sex**	**Sample collection date**	**Time of Assessment**	**Date disease initiated**	**Follow up date**	**Treatment**	**Date of BCR-ABL detection**	**BCR-ABL%**	**Comments based on BCR-ABL**
64	14	35/F	29-05-2014	New	25-05-2014	04-05-2015	Imatinib	09-06-2014	85.56	
65	19	28/M	29-05-2014	New	15-05-2014	22-12-2014	Imatinib	15-05-2014	100	Imatinib non-sensitive switched to nilotinib
66	34	24/M	12-06-2014	New	20-06-2014	22-07-2015	Imatinib and since February 2015 Nilotinib	20-06-2014	35.63	Imatinib non-sensitive
								27-11-2014	95.37	
67	8	24/M	22-05-2014	8 months	26-09-2013	15-05-2014	Imatinib	26-09-2013	100	Imatinib non-sensitive
								20-06-2014	26.89	
68	29	35F	05-06-2014	1 month	04-05-2014		Imatinib	04-05-2014	89.12	Imatinib non-sensitive
								01-08-2014	15.89	
69	47	48/M	17-07-2014	3 months	17-07-2014	29-04-2015	Imatinib and 29-05-2014 Uridrea	27-07-2014	40.12	Imatinib non-sensitive
								29-10-2014	32.45	
70	49	15/M	31-07-2014	1.4 yrs	09-05-2013	11-05-2015	Imatinib	06-03-2013	100	Imatinib non-sensitive
								30-06-2014	not detected	

Whole blood collected from 70 patients enrolled in this study were subjected to DNA and RNA extraction using QIAamp DNA mini kit (Qiagen, Hilden, Germany) and Trizol, respectively. The quality and quantity of DNA was checked both using a Quawell- spectrophotometer (Quawell Technology Inc., San Jose, CA 95161-2738) and a QubitBR-Fluorimeter (Agilent, Santa Clara, CA, USA). DNA samples with an absorption ratio (A260/A280) greater than 1.9 was considered for further CNV analysis using the Molecular Inversion based probe array (MIP-based array). RNA quantity and purity were determined by using the Samples with purity ratios (A260/A280) between 1.80 and 2.00 were considered for further analyses. Formaldehyde agarose gel were used to check the integrity of the extracted RNA; only samples with a 2:1 ratio of the 18S and 28S ribosomal RNAs were used for further transcriptomic analysis using the human transcriptome array 2.0 [51, 52].

### Molecular inversion probe (MIP)-based oncoscan array hybridization

Using the MIP-based Oncoscan array, 34 CML (out of 70 CML) and 5 control samples (one male, two females, and kit-derived positive and negative controls) were processed for CNV profiling using 12 ng/μL DNA per sample. According to the recommended protocol, the chips were processed and scanned through GENECHIP Scanner-7G (Affymetrix, CA) for identification of copy number and somatic mutation variations as reported previously [51]. Further, the OSCHP file generated through Oncoscan Console Software (Biodiscovery, Inc., CA USA) was analyzed via Tumor Scan (TuScan) and BioDiscovery's SNP-FASST2 algorithm using the Nexus Express for Oncoscan software version 7.5 (Biodiscovery, Inc., CA USA). The TuScan algorithm creates segmentation to differentiate between adjacent clusters of probes and determines copy number variations. The BioDiscovery's SNP-FASST2 algorithm, a proprietary variation of a Hidden Markov Model (HMM), is used to identify allelic event calls.

### Human transcriptome array 2.0 hybridization

For transcriptomics, we processed 35 CML samples (out of 70 CML-samples) and 4 control samples. The CEL files were generated by processing 500 ng of total RNA on Affymetrix HTA 2.0 arrays according to the manufacturer's recommendations (Affymetrix, Santa Clara, Calif) and were scanned through GENECHIP Scanner-7G (Affymetrix, CA) [52]. The CEL files generated by these arrays were converted into rma-gene-ful.chp and. rma-alt-splice-dabg.chp files through Affymetrix Expression Console™ Software (version 1.3). The data was then analyzed through Transcriptome Analysis Consolev3.0. After running ANOVA, multi-testing correction was performed using Benjamini-Hochberg Step-Up FDR-controlling procedure for all expressed genes and expressed PSRs/Junctions (i.e., expressed in at least one condition). By default, the alpha level was set at 0.05 in the false discovery rate field.

### Biobank genotyping array hybridization

Axiom Biobank genotyping arrays was used to genotype 65 CML-TKI-treated CML cases- (out of 70 CML-samples) and 30 control samples (Supplementary Table 3). Out of 65 cases, 19 new samples-Imatinib treated (CML-CP-UT) with BCR-ABL ranging from 10 to 77.02% (numbered 16, 50, 52-54, 60, 61, 66, 67, 75-82, 84, 85 and 88) were included in the study but were not processed for CNVs and Transcriptomics. The experiment was conducted as recommended by the manufacturer's protocol. All processed samples passed dish quality control (DQC), quality control call rate (QC CR) and plate quality control (QC). Average quality control (QC) call rate for the passing samples was 99.571.

### REAL TIME SNP genotyping

We designed unlabeled PCR primers and TaqManMGBprobes (FAM and VIC dye-labeled) in 40X assay mix (Assays-by-DesignSM Service for SNP Genotyping Assays) to genotype rs2854344 and rs9475077. Alleles were scored in each well using TaqMan Genotyping Master Mix and 20 ng of specific genomic DNA following the universal thermal cycling parameters per the recommended protocol. Each sample was processed in triplicate, and a negative control was also processed for real-time analysis with every 96-well format assay. The raw data were obtained using ABI Step OnePlus Real-Time PCR System and were analyzed through TaqMan Genotype software. The genotype call was evaluated with a threshold quality value=0.94.

### BCR-ABL transcript determination

For the quantitative detection and differentiation of BCR-ABL fusion gene transcripts major (M), minor (m), and micro (μ) in peripheral blood samples from CML patients, TRUPCR BCR-ABL REAL TIME PCR KIT was used with a real-time PCR system (ABI Step one plus). This kit is designed according to the “Europe Against Cancer” (EAC) and Guidelines for the measurement of BCR-ABL transcripts in CML patients with the updated international recommendations. It has a two-step protocol in which total RNA is reverse-transcribed, and the generated cDNA is amplified by PCR using a pair of specific primers and a specific internal double-dye probe for BCR-ABL (major, minor, and micro) and ABL. A standard curve was plotted against a known number of copies of BCR-ABL 1 and ABL1. Normalized copy number (NCN) was calculated using the following formula: NCN(%)= (BCR-ABL CN/ABL1 CN)^*^100.

### Expression analysis of selected genes using quantitative real-time PCR

Comparative relative expression of FOS, TGFBR2, TPX2, LAPTM4B, HLTF, CFH, PIEZO2, CD109, ANGPT1 against β actin and 18s ribosomal reference gene were measured by real-time PCR (RT-PCR) in 23 CML and 8 control samples. The RT-PCR amplifications were carried out using a ABI Stepone RT-PCR in a final volume of 20 μl containing 0.5 μl normalized cDNA, 10 pmol of each primer and 10 μl SYBR green master mix together with a negative control with no template by following RT-PCR steps; activation step at 95 °C for 5 min, followed by 40 cycles of: 15 s at 95 °C, 15 s at the Tm specific for the primer pairs used, and 35 s at 72 °C with a single fluorescence measurement. After the amplification phase, a melting curve cycle was set at 95 °C for 5 s, 67 °C for 1 min with acquisitions 5 per °C from 97 to 65 °C and a continuous measurement was performed to confirm later about the amplification of a single product. RT-PCR was repeated twice for each sample in triplicates. The crossing point, Ct values was acquired for both the target and reference gene using ABI Stepone RT-PCR software. The relative level of each transcript in different tissue was calculated by normalization of the value with the corresponding reference and compared among them using Ct values for tumor cDNA as positive calibrator. Comparison of relative expression level of each transcript was analyzed by REST 2009 software with 2000 time iterations (http://www.REST.de.com).

## CONCLUSIONS

The highly significant down-regulation of STAT6, FOS, TGFBR2, and ITK and up-regulation of MPO, TPX2, and TYMS in drug-treated CML cases relative to normal samples led to the up-regulation of cell cycle, DNA replication, and DNA repair pathways and down-regulation of immune system, chemokine and interleukin signaling, TCR signaling, TGF beta signaling, and MAPK signaling pathways. Further, significant up-regulation of LAPTM4B, HLTF, PIEZO2, CFH, CD109, ANGPT1 influence autophagy, stem cell, complement system, TGFβ Receptor and homeostasis pathway subsequently leading to resistance in >1% BCR-ABL copies of CML treated cases.

Hence, we suggest that genes included in these pathways may be used as markers for CML development (FOS, TGFBR2, TPX2) and CML resistance to therapy (LAPTM4B, HLTF, PIEZO2, CFH, CD109, ANGPT1). This dynamic was accompanied by a loss of 7q31.2 (MET) in low CNVs in the undetectable BCR-ABL-TKI-sensitive group and were identified and previously reported as CNVs 9q34.11-q34.2 (FNBP1, ABL1, NUP214, TSC1, RALGDS) in the high CNVs-BCR-ABL-dependent and independent-TKI-non-sensitive groups. Further, rsID239798 (Ch6:54940890) and rsID9475077 (Ch6:54941691) were associated with FAM83B, a proto-oncogene that has previously been implicated in therapeutic resistance to TKI.

## SUPPLEMENTARY MATERIALS FIGURES AND TABLES










