# Supplementary material for: Differential genomics and transcriptomics between tyrosine kinase inhibitor-sensitive and -resistant BCR-ABL-dependent chronic myeloid leukemia
Source: Oncotarget. 2018 Jul 13;9(54):30385–418. doi: 10.18632/oncotarget.25752 (PMC6084383; doi:10.18632/oncotarget.25752)
Supplement: Supplementary file 1 [file oncotarget-09-30385-s001.pdf]

Differential genomics and transcriptomics between tyrosine kinase inhibitor-sensitive and -resistant BCR-ABL-dependent chronic myeloid leukemia

SUPPLEMENTARY MATERIALS

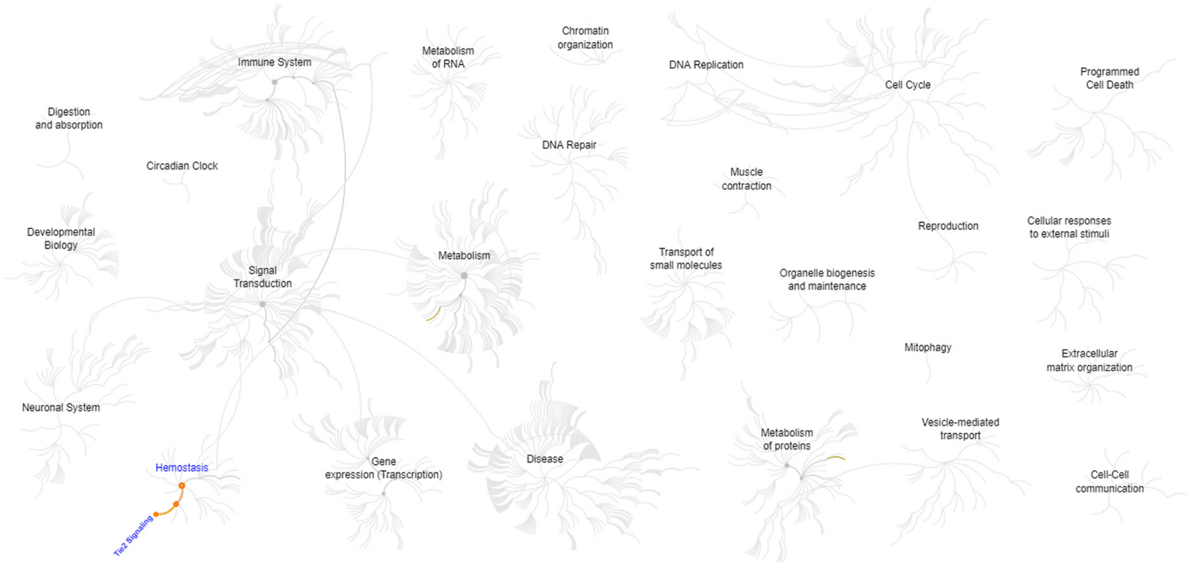

Supplementary Figure 1: Over-expression of hemostasis pathway with specific involvement of Tie2 and basigin-transmembrane glycoprotein signaling pathway through Reactome Pathway analysis software.

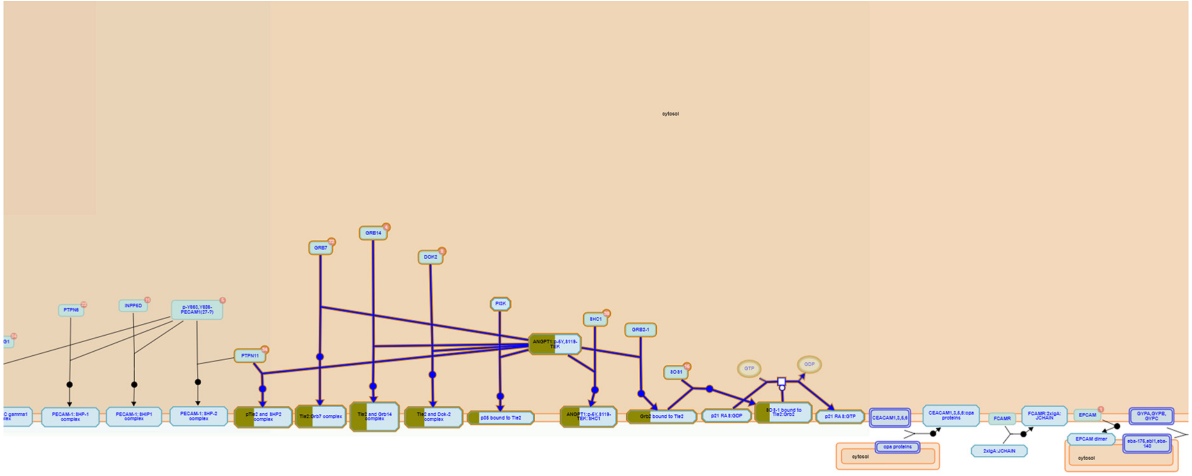

Supplementary Figure 2: Over-expression of hemostasis pathway with specific involvement of Tie2 signaling.

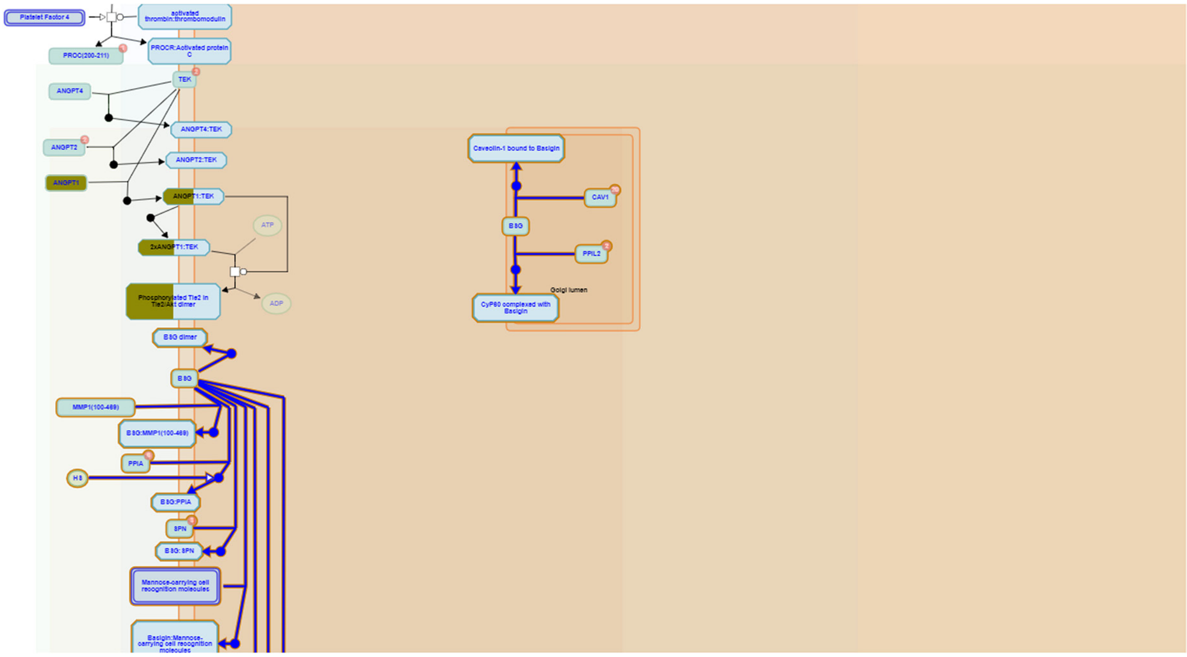

Supplementary Figure 3: Over-expression of hemostasis pathway with specific involvement of basigin-transmembrane glycoprotein signalling pathway.

Supplementary Figure 4a

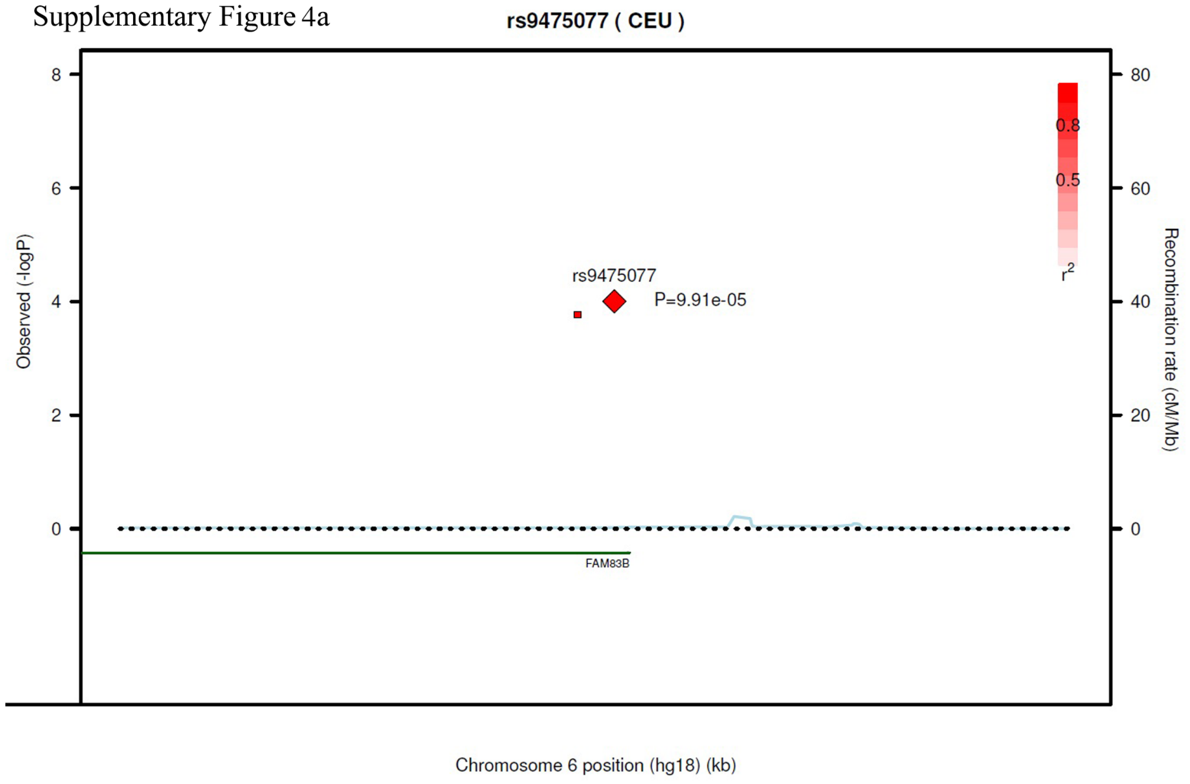

Supplementary Figure 4b

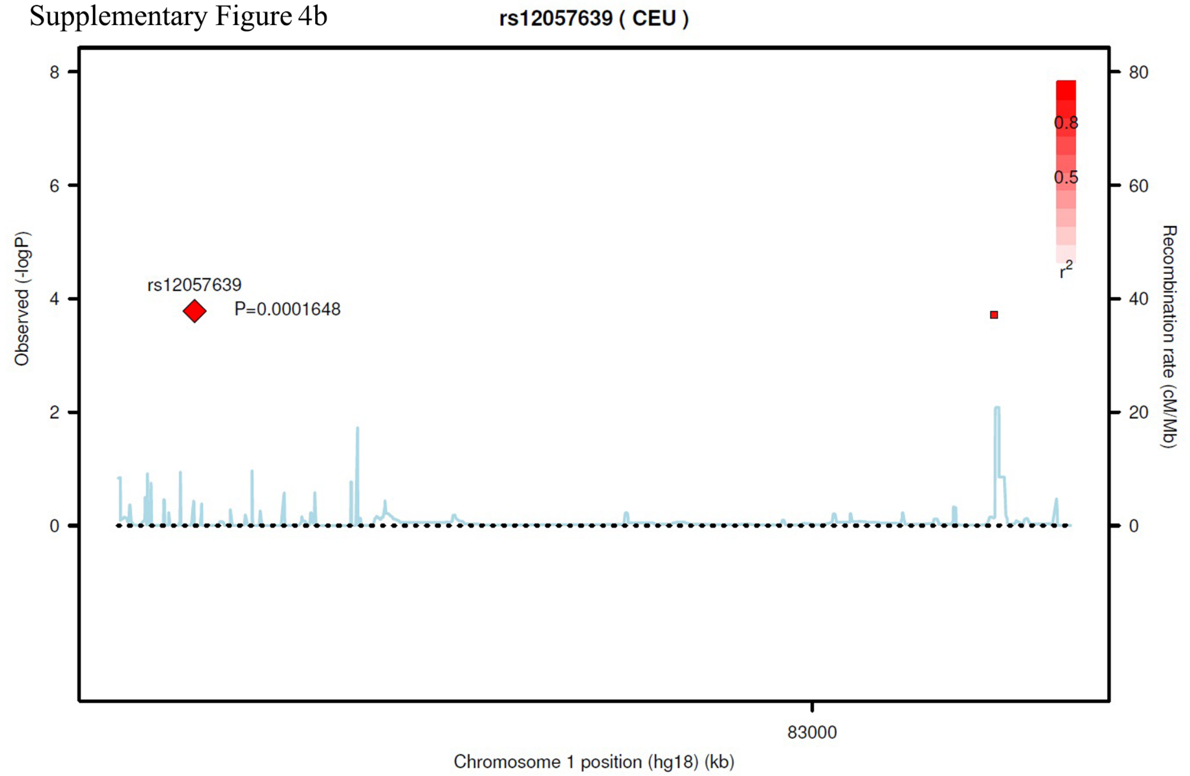

Supplementary Figure 4: (a) Association plot for query and proxy SNP rs12057639 and rs1327107 with r2=0.934 (TIF). **(b)** Association plot for query and proxy SNP rs239798 and rs9475077 with r2=1(TIF).

Supplementary Table 1: Catalogue of differentially expressed gene with (< or > 2 fold; p=0.01; default FDR p-value) with significant fold change (linear) considering only on the basis of NM IDs in all CML versus control samples (XLSX)

See Supplementary File 1

Supplementary Table 2a: Differential regulation of pathways in CML compared to control at p=0.01

See Supplementary File 2

Supplementary Table 2b: Differential regulation of pathways in CML compared to control at p=0.01 and FDR p value=0.05

See Supplementary File 2

Supplementary Table 3a: Genes myeloperoxidase (MPO), thymidylate synthetase (TYMS) and TPX2, microtubule associated (TPX2) up-regulated 121.81-, 10.12- and 7.56-fold respectively at exon level, among CML cases when subjected to Reactome Pathway database specifically up-regulated cell cycle pathways and other enlisted pathways

See Supplementary File 3

Supplementary Table 3b: While, genes IL-2-inducible T cell kinase (ITK), FBJ murine osteosarcoma viral oncogene homolog (FOS), and src kinase associated phosphoprotein 1 (SKAP1) down-regulated 7.59-, 15.22- and 5.09-fold, respectively, among CML samples and showed down-regulation of the immune system and other enlisted pathways

See Supplementary File 3

Supplementary Table 4: Sample QC data for genotyping array sample QC data for genotyping array (XLSX)

See Supplementary File 4

Supplementary Table 5a: Associations between individual SNP and CML-risk were assessed using p=0.001 and odds ratios (ORs) > 4.0 and 95% confidence intervals (CIs) derived from logistic regression models (XLSX)

CHR	SNP ID	Pysical Position (base pair)	Minor allele name (based on whole sample-A1)	Frequency of this allele (A) in cases	Frequency of this allele (A) in controls	Major allele name (A2)	Basic allelic test chi-square (1df)-CHISQ	Asymptotic p-value for this test (P)	Estimated odds ratio (for A1, i.e. A2 is reference)-OR	SE	Lower bound of 95% confidence interval for odds ratio (L95)	Upper bound of 95% confidence interval for odds ratio (U95)
1	rs12057639	83147308	G	0.4385	0.1613	A	14.2	0.000165	4.06	0.3879	1.898	8.684
1	rs1327107	83250957	T	0.4154	0.1452	G	13.9	0.000192	4.184	0.4021	1.903	9.201
2	rs1466556	22896073	T	0.4054	0.02778	C	17.06	3.62E-05	23.86	1.041	3.099	183.7
2	rs61137390	239637690	G	0.2462	0.01613	A	15.61	7.80E-05	19.92	1.029	2.653	149.5
4	rs11933386	106812681	A	0.3308	0.08065	C	14.01	0.000182	5.634	0.5023	2.105	15.08
5	rs7444183	107865111	C	0.2969	0.04839	T	15.24	9.47E-05	8.304	0.6227	2.451	28.14
6	rs239798	54805688	C	0.2578	0.03226	A	14.14	0.00017	10.42	0.7467	2.412	45.03
6	rs9475077	54806489	A	0.2692	0.03226	C	15.15	9.91E-05	11.05	0.7455	2.564	47.65
6	rs1342840	91596456	A	0.4154	0.1452	G	13.9	0.000192	4.184	0.4021	1.903	9.201
6	rs12193041	132338996	A	0.3231	0.04839	G	17.65	2.65E-05	9.386	0.6209	2.78	31.69
12	rs73142055	78293160	T	0.4804	0.12	C	18.87	1.40E-05	6.78	0.4782	2.656	17.31
13	rs74932235	26612455	G	0.2734	0.03226	A	15.49	8.28E-05	11.29	0.7456	2.618	48.69
13	rs10507360	26616294	T	0.2615	0.03226	C	14.49	0.000141	10.62	0.746	2.462	45.85
16	rs4077730	7877054	C	0.3538	0.09677	T	14.05	0.000178	5.111	0.4671	2.046	12.77
16	rs8050297	53054491	G	0.4919	0.1897	A	15.1	0.000102	4.137	0.3801	1.964	8.714
20	rs208817	37492316	C	0.3692	0.09677	T	15.42	8.63E-05	5.463	0.4664	2.19	13.63
21	rs2825294	20404094	A	0.3154	0.06452	G	14.72	0.000125	6.68	0.5503	2.272	19.64

Supplementary Table 5b: Identification of Proxy-SNPs (XLSX)

SNP	Proxy	Distance	RSquared	DPrime	Arrays	Chromosome	Coordinate_HG18
rs12057639	rs1327107	103649	0.934	1	AAE	chr1	83023545
rs1327107	rs12057639	103649	0.934	1	AS,A5,A6,IM,IMD,OQ,OE,O24,O28,O54,O5E,OEE,AAE	chr1	82919896
rs239798	rs9475077	801	1	1	AG,A6,I1,IM,IMD,OQ,AxM,OE,O24,O28,O54,O5E,OEE,AAE	chr6	54914448
rs9475077	rs239798	801	1	1	AG,I1,I3,I5,I6,I6Q,IM,IMD,IC,ICQ,OQ,IWQ,OE,O24,O28,O54,O5E,OEE,AAE	chr6	54913647
