# Supplementary material for: Differential genomics and transcriptomics between tyrosine kinase inhibitor-sensitive and -resistant BCR-ABL-dependent chronic myeloid leukemia
Source: Oncotarget. 2018 Jul 13;9(54):30385–418. doi: 10.18632/oncotarget.25752 (PMC6084383; doi:10.18632/oncotarget.25752)
Supplement: Supplementary file 3 [file oncotarget-09-30385-s003.docx]

**Supplementary Table 2a :** Differential regulation of pathways in CML compared to Control at p=0.01. Some pathways of significance (calculated using a 2x2 contingency in a Fisher's Exact Test (Two Sided)) like Retinoblastoma (RB) in Cancer, Cell Cycle, DNA Replication etc. were up-regulated and TCR, Chemokine Signaling pathways etc. were down-regulated in CML compared to control. These pathways were identified through WikiPathways (wikipathways.org).

| **Pathway** | **#Total** | **#Up** | **Up List** | **#Down** | **Down List** | **Significance** | **p-value** |
| --- | --- | --- | --- | --- | --- | --- | --- |
| Retinoblastoma (RB) in Cancer | 25 | 24 | CCNA2,CCNE2,CDK1,PLK4,CCNB2,MCM6,RFC4,TOP2A,RFC3,MCM4,POLA1,ORC1,CHEK1,PCNA,BARD1,TTK,KIF4A,RRM1,RRM2,HMGB2,TYMS,ANLN,CDC7,WEE1 | 1 | CDC25B | 24.44 | 0 |
| Cell Cycle | 16 | 14 | BUB1B,BUB1,WEE1,CCNA2,PCNA,CDK1,CCNB2,CDC7,CDC6,CCNE2,MCM4,MCM6,MCM10,ORC1 | 2 | ATM,MPEG1 | 11.53 | 0 |
| DNA Replication | 8 | 8 | RFC4,PCNA,CDC7,MCM6,RFC3,MCM10,MCM4,CDC6 | 0 |  | 6.8 | 0 |
| DNA IR-damage and cellular response via ATR | 10 | 9 | RAD51,FANCI,PCNA,CLSPN,EXO1,DCLRE1A,BRIP1,BARD1,FOXM1 | 1 | ATM | 6.4 | 0 |
| Allograft Rejection | 10 | 0 |  | 10 | CD28,CD40LG,CTLA4,HLA-DRB3,HLA-DRB1,HLA-DQA2,HLA-DOB,HLA-DQA1,HLA-DQB1,HLA-DMA | 5.98 | 0.000001 |
| Gastric Cancer Network 2 | 6 | 6 | UBE2T,FANCI,RFC3,TOP2A,ATAD2,RFC4 | 0 |  | 5.19 | 0.000006 |
| T-Cell antigen Receptor (TCR) Signaling Pathway | 9 | 0 |  | 9 | FOS,ITK,CD3E,CD3D,PLCG1,CD4,CD28,SKAP1,ICOS | 5.06 | 0.000009 |
| Vitamin D Receptor Pathway | 12 | 0 |  | 12 | TREM1,HLA-DRB1,HLA-DQA1,CTLA4,RASGRP1,BTLA,CD14,SLC8A1,MX2,JUNB,ADRB2,FOXO1 | 4.66 | 0.000022 |
| Histone Modifications | 7 | 7 | HIST1H3D,EZH2,HIST1H4C,HIST1H4L,HIST1H3F,HIST1H3B,HIST1H4D | 0 |  | 4.24 | 0.000058 |
| Gastric Cancer Network 1 | 5 | 5 | CENPF,MCM4,ECT2,TOP2A,TPX2 | 0 |  | 4.22 | 0.00006 |
| G1 to S cell cycle control | 7 | 6 | CCNE2,WEE1,MCM4,MCM6,PCNA,CDK1 | 1 | ATM | 4.15 | 0.00007 |
| Chemokine signaling pathway | 10 | 0 |  | 10 | CCR6,CCR4,CCR7,CX3CR1,XCL1,CXCL16,JAK3,LYN,ITK,TIAM1 | 3.73 | 0.000186 |
| Mitotic G1-G1/S phases | 12 | 12 | WEE1,MCM10,CDC6,PCNA,TOP2A,CDK1,POLA1,ORC1,FBXO5,RRM2,TYMS,CCNA2 | 0 |  | 3.6 | 0.000251 |
| Spinal Cord Injury | 8 | 2 | ARG1,CDK1 | 6 | TNFSF13B,LTB,ZFP36,BTG2,FOS,OMG | 3.46 | 0.000348 |
| Hair Follicle Development: Cytodifferentiation (Part 3 of 3) | 7 | 1 | ELANE | 6 | FOS,DSC2,BCL11B,FOSB,LEF1,BMPR1A | 3.4 | 0.000399 |
| TGF-beta Signaling Pathway | 8 | 2 | CCNB2,CDK1 | 6 | FOS,FOSB,TGFBR2,ETS1,JUNB,LIMK2 | 3.05 | 0.0009 |
| T-Cell Receptor and Co-stimulatory Signaling | 4 | 0 |  | 4 | PLCG1,ITK,CD28,CTLA4 | 2.97 | 0.001064 |
| Endoderm Differentiation | 8 | 4 | WDHD1,NCAPG2,HPRT1,EZH2 | 4 | TCF7,FOXO1,BMPR1A,LEF1 | 2.85 | 0.001425 |
| Fluoropyrimidine Activity | 4 | 4 | TYMS,RRM1,GGH,RRM2 | 0 |  | 2.82 | 0.001515 |
| Amplification and Expansion of Oncogenic Pathways as Metastatic Traits | 3 | 0 |  | 3 | CYTIP,TCF7,LEF1 | 2.73 | 0.001849 |
| TYROBP Causal Network | 5 | 1 | IL18 | 4 | CXCL16,CD4,RBM47,IL10RA | 2.72 | 0.001898 |
| Simplified Depiction of MYD88 Distinct Input-Output Pathway | 3 | 0 |  | 3 | TLR1,TLR5,TLR10 | 2.66 | 0.002194 |
| Hematopoietic Stem Cell Differentiation | 6 | 1 | MYB | 5 | FOSB,LEF1,TRAF3IP3,FOS,PIM2 | 2.62 | 0.002416 |
| Nucleotide Metabolism | 3 | 3 | HPRT1,RRM1,RRM2 | 0 |  | 2.59 | 0.002578 |
| EGF/EGFR Signaling Pathway | 8 | 1 | PCNA | 7 | FOS,FOSB,LIMK2,PLCG1,MAP3K1,FOXO1,RICTOR | 2.54 | 0.002851 |
| DNA Damage Response | 5 | 4 | CHEK1,CCNE2,CCNB2,RAD51 | 1 | ATM | 2.48 | 0.003293 |
| miRNA Regulation of DNA Damage Response | 6 | 5 | CHEK1,CCNE2,CDK1,CCNB2,RAD51 | 1 | ATM | 2.48 | 0.003314 |
| B Cell Receptor Signaling Pathway | 6 | 0 |  | 6 | PLCG1,FOXO1,LYN,ETS1,CD79A,CD22 | 2.44 | 0.003663 |
| Arrhythmogenic Right Ventricular Cardiomyopathy | 5 | 0 |  | 5 | SLC8A1,DSC2,ITGB7,LEF1,TCF7 | 2.32 | 0.004738 |
| IL1 and megakaryocytes in obesity | 3 | 1 | IL18 | 2 | PLA2G7,TLR1 | 2.29 | 0.005099 |
| IL-7 Signaling Pathway | 3 | 0 |  | 3 | IL2RG,IL7R,JAK3 | 2.24 | 0.005732 |
| Regulation of toll-like receptor signaling pathway | 7 | 0 |  | 7 | TLR1,TLR5,CD180,CD14,IFNAR1,TREM1,FOS | 2.17 | 0.00671 |
| Mismatch repair | 2 | 2 | PCNA,EXO1 | 0 |  | 2.14 | 0.007273 |
| Macrophage markers | 2 | 0 |  | 2 | CD14,CD74 | 2.14 | 0.007273 |
| Macrophage markers | 2 | 0 |  | 2 | CD14,CD74 | 2.14 | 0.007273 |
| Pyrimidine metabolism | 5 | 5 | TYMS,DUT,RRM2,RRM1,POLA1 | 0 |  | 2.07 | 0.008466 |
| Signaling by Interleukins | 5 | 1 | IL18 | 4 | CD4,IL32,CSF1R,IL2RG | 2.07 | 0.008466 |
| PDGFR-beta pathway | 3 | 0 |  | 3 | PLCG1,STAT6,FOS | 2.06 | 0.00872 |
| Other interleukin signaling | 4 | 1 | IL18 | 3 | CD4,IL32,CSF1R | 1.98 | 0.010518 |
| DNA IR-Double Strand Breaks (DSBs) and cellular response via ATM | 4 | 3 | RAD51,EXO1,PCNA | 1 | ATM | 1.98 | 0.010518 |
| Kit receptor signaling pathway | 4 | 0 |  | 4 | FOS,PLCG1,LYN,JUNB | 1.95 | 0.011155 |
| Inflammatory Response Pathway | 3 | 0 |  | 3 | IL2RG,CD28,CD40LG | 1.94 | 0.011459 |
| White fat cell differentiation | 3 | 0 |  | 3 | FOXO1,TLE3,KLF2 | 1.94 | 0.011459 |
| White fat cell differentiation | 3 | 0 |  | 3 | FOXO1,TLE3,KLF2 | 1.94 | 0.011459 |
| Serotonin HTR1 Group and FOS Pathway | 3 | 0 |  | 3 | RASGRP1,MAP3K1,FOS | 1.9 | 0.012469 |
| Corticotropin-releasing hormone signaling pathway | 5 | 1 | IL18 | 4 | JUNB,FOS,FOSB,PLCG1 | 1.88 | 0.013288 |
| Focal Adhesion-PI3K-Akt-mTOR-signaling pathway | 10 | 0 |  | 10 | JAK3,ITGB7,PIK3IP1,IFNAR1,IL7R,ITGAL,IL2RG,CSF1R,FOXO1,CREB5 | 1.83 | 0.014631 |
| Homologous recombination | 2 | 1 | RAD51 | 1 | ATM | 1.82 | 0.015157 |
| GPCRs, Class A Rhodopsin-like | 9 | 1 | SUCNR1 | 8 | ADRB2,CCR4,CCR6,CX3CR1,CCR7,P2RY10,PTAFR,GPR15 | 1.79 | 0.016154 |
| Oncostatin M Signaling Pathway | 4 | 0 |  | 4 | JAK3,FOS,JUNB,RICTOR | 1.79 | 0.016311 |
| Toll-like Receptor Signaling Pathway | 5 | 0 |  | 5 | TLR1,TLR5,CD14,IFNAR1,FOS | 1.75 | 0.01759 |
| GPR40 Pathway | 2 | 0 |  | 2 | PLCL1,PLCG1 | 1.7 | 0.020012 |
| Brain-Derived Neurotrophic Factor (BDNF) signaling pathway | 6 | 0 |  | 6 | PLCG1,TIAM1,CAMK4,MAP3K1,FOS,ALPL | 1.67 | 0.021336 |
| ATM Signaling Pathway | 3 | 2 | CDK1,RAD51 | 1 | ATM | 1.65 | 0.022355 |
| Peptide GPCRs | 4 | 0 |  | 4 | CCR7,CX3CR1,CCR4,CCR6 | 1.62 | 0.023782 |
| IL-2 Signaling Pathway | 3 | 0 |  | 3 | IL2RG,FOS,JAK3 | 1.62 | 0.023819 |
| Collagen degradation | 1 | 1 | ELANE | 0 |  | 1.62 | 0.023883 |
| IL-9 Signaling Pathway | 2 | 0 |  | 2 | JAK3,IL2RG | 1.59 | 0.025425 |
| Mitotic Telophase/Cytokinesis | 2 | 2 | KIF23,KIF20A | 0 |  | 1.55 | 0.028329 |
| MAPK Signaling Pathway | 8 | 0 |  | 8 | DUSP1,RASGRP1,CD14,MAP3K1,RASGRF2,CDC25B,FOS,TGFBR2 | 1.53 | 0.02941 |
| Exercise-induced Circadian Regulation | 3 | 1 | HIST1H2BN | 2 | HLA-DMA,ARNTL | 1.47 | 0.033658 |
| Toll-Like Receptors Cascades | 4 | 1 | BPI | 3 | TLR10,CD14,TLR5 | 1.47 | 0.033658 |
| Thymic Stromal LymphoPoietin (TSLP) Signaling Pathway | 3 | 0 |  | 3 | IL7R,STAT6,LYN | 1.47 | 0.033658 |
| PI3K-Akt Signaling Pathway | 10 | 2 | CCNE2,MYB | 8 | JAK3,ITGB7,IFNAR1,IL7R,IL2RG,CSF1R,CREB5,SGK1 | 1.44 | 0.036346 |
| Photodynamic therapy-induced AP-1 survival signaling. | 3 | 1 | CCNA2 | 2 | JUNB,FOS | 1.43 | 0.037339 |
| Imatinib and Chronic Myeloid Leukemia | 2 | 0 |  | 2 | CSF1R,PIM2 | 1.42 | 0.037778 |
| Integrated Breast Cancer Pathway | 6 | 3 | WEE1,RAD51,BARD1 | 3 | ATM,FOXO1,BMPR1A | 1.41 | 0.038588 |
| The human immune response to tuberculosis | 2 | 0 |  | 2 | PSMB8,IFNAR1 | 1.39 | 0.041159 |
| Kinesins | 3 | 3 | KIF11,RACGAP1,KIF18A | 0 |  | 1.38 | 0.041216 |
| TGF-beta Receptor Signaling | 3 | 0 |  | 3 | TGFBR2,LEF1,FOS | 1.32 | 0.047396 |
| RANKL/RANK (Receptor activator of NFKB (ligand)) Signaling Pathway | 3 | 0 |  | 3 | FOS,LYN,PLCG1 | 1.32 | 0.047396 |

**Supplementary Table 2b :** Differential regulation of pathways in CML compared to Control at p=0.01 and FDR p value=0.05. TGFBR2, FOS, PLCL1, STAT6, CHMP7 and SIRPG down-regulated the enlisted pathways. These pathways were identified through WikiPathways (wikipathways.org).

| **Pathway** | **#Total** | **#Up** | **Up List** | **#Down** | **Down List** | **Significance** | **p-value** |
| --- | --- | --- | --- | --- | --- | --- | --- |
| TGF-beta Receptor Signaling | 2 | 0 |  | 2 | TGFBR2,FOS | 3.21 | 0.000618 |
| PDGFR-beta pathway | 2 | 0 |  | 2 | STAT6,FOS | 3.77 | 0.000171 |
| MAPK Signaling Pathway | 2 | 0 |  | 2 | FOS,TGFBR2 | 1.94 | 0.011546 |
| TGF-beta Signaling Pathway | 2 | 0 |  | 2 | FOS,TGFBR2 | 2.44 | 0.003649 |
| Spinal Cord Injury | 2 | 0 |  | 2 | FOS,OMG | 2.56 | 0.002758 |
| Serotonin HTR1 Group and FOS Pathway | 1 | 0 |  | 1 | FOS | 1.66 | 0.021923 |
| IL-2 Signaling Pathway | 1 | 0 |  | 1 | FOS | 1.56 | 0.027822 |
| Endometrial cancer | 1 | 0 |  | 1 | FOS | 1.38 | 0.041458 |
| Oxidative Stress | 1 | 0 |  | 1 | FOS | 1.7 | 0.019949 |
| GPR40 Pathway | 1 | 0 |  | 1 | PLCL1 | 2 | 0.010022 |
| IL-4 Signaling Pathway | 1 | 0 |  | 1 | STAT6 | 1.43 | 0.037581 |
| Serotonin and anxiety | 1 | 0 |  | 1 | FOS | 1.94 | 0.011351 |
| Serotonin and anxiety-related events | 1 | 0 |  | 1 | FOS | 2.06 | 0.008691 |
| Canonical and Non-Canonical TGF-B signaling | 1 | 0 |  | 1 | TGFBR2 | 1.94 | 0.011351 |
| Hypothesized Pathways in Pathogenesis of Cardiovascular Disease | 1 | 0 |  | 1 | TGFBR2 | 1.78 | 0.01665 |
| Photodynamic therapy-induced NFE2L2 (NRF2) survival signaling | 1 | 0 |  | 1 | FOS | 1.81 | 0.015328 |
| Photodynamic therapy-induced AP-1 survival signaling. | 1 | 0 |  | 1 | FOS | 1.48 | 0.033038 |
| Sleep regulation | 1 | 0 |  | 1 | FOS | 1.6 | 0.025205 |
| Endosomal Sorting Complex Required For Transport (ESCRT) | 1 | 0 |  | 1 | CHMP7 | 1.56 | 0.027822 |
| Signaling of Hepatocyte Growth Factor Receptor | 1 | 0 |  | 1 | FOS | 1.65 | 0.02258 |
| Kit receptor signaling pathway | 1 | 0 |  | 1 | FOS | 1.41 | 0.038875 |
| Extracellular vesicle-mediated signaling in recipient cells | 1 | 0 |  | 1 | TGFBR2 | 1.7 | 0.019949 |
| IL-3 Signaling Pathway | 1 | 0 |  | 1 | FOS | 1.49 | 0.032388 |
| Selenium Metabolism and Selenoproteins | 1 | 0 |  | 1 | FOS | 1.54 | 0.029129 |
| Signaling by TGF-beta Receptor Complex | 1 | 0 |  | 1 | TGFBR2 | 1.31 | 0.049168 |
| Integrated Lung Cancer Pathway | 1 | 0 |  | 1 | FOS | 1.54 | 0.029129 |
| Quercetin and Nf-kB/ AP-1 Induced Cell Apoptosis | 1 | 0 |  | 1 | FOS | 1.97 | 0.010687 |
| Oncostatin M Signaling Pathway | 1 | 0 |  | 1 | FOS | 1.36 | 0.043391 |
| Thymic Stromal LymphoPoietin (TSLP) Signaling Pathway | 1 | 0 |  | 1 | STAT6 | 1.5 | 0.031737 |
| Human Thyroid Stimulating Hormone (TSH) signaling pathway | 1 | 0 |  | 1 | FOS | 1.36 | 0.043391 |
| RANKL/RANK (Receptor activator of NFKB (ligand)) Signaling Pathway | 1 | 0 |  | 1 | FOS | 1.44 | 0.036285 |
| miR-targeted genes in adipocytes - TarBase | 1 | 0 |  | 1 | TGFBR2 | 1.59 | 0.02586 |
| Signal regulatory protein family interactions | 1 | 0 |  | 1 | SIRPG | 1.81 | 0.015328 |
| MAPK targets/ Nuclear events mediated by MAP kinases | 1 | 0 |  | 1 | FOS | 1.49 | 0.032388 |
| Physiological and Pathological Hypertrophy of the Heart | 1 | 0 |  | 1 | FOS | 1.78 | 0.01665 |
| IL-5 Signaling Pathway | 1 | 0 |  | 1 | FOS | 1.57 | 0.027169 |
